# Predicting pore-carrier solubility and size-exclusivity towards the rational design of type II porous liquid solutions†

**DOI:** 10.1039/d5sc01875g

**Published:** 2025-05-21

**Authors:** Austin M. Mroz, Benjamin D. Egleston, James Sherwood, Ruby C. Morel, Kim E. Jelfs, Rebecca L. Greenaway

**Affiliations:** a Department of Chemistry, Molecular Sciences Research Hub, Imperial College London White City Campus, 82 Wood Lane W12 0BZ UK; b I-X Centre for AI in Science, Imperial College London, White City Campus 84 Wood Lane London W12 0BZ UK; c Green Chemistry Centre of Excellence, University of York Heslington York Yorkshire YO10 5DD UK; d Department of Chemistry and Materials Innovation Factory, University of Liverpool 51 Oxford Street Liverpool L7 3NY UK a.mroz@imperial.ac.uk r.greenaway@imperial.ac.uk

## Abstract

Porous liquids are a sub-class of porous materials that combine permanent porosity, typically associated with solids, with the fluidity and fast mass-transfer capabilities of liquids, making them ideal candidates for gas storage and separation applications. One strategy to form porous liquids is the dissolution of discrete and permanently porous molecular species at relatively high concentrations in cavity-excluded solvents, thus introducing permanent porosity into the liquid in which it is dissolved and ensuring a solution of reasonable porosity is obtained. To access high-performance porous liquids for target applications, the selection of both the porous molecular species and the cavity-excluded solvent is key to ensuring the solvent is permanently excluded and the pore carrier is highly soluble. Finding new solvents that fit both these requirements is challenging, often resulting in a trial-and-error approach. While predictive data-driven models may be attractive, the youth of the porous liquid field currently limits the availability of data necessary to train robust models. Here, we present a computational workflow for the discovery of new porous liquid solutions combining solubility prediction software and a size-exclusivity prediction algorithm that correctly predicts size-exclusivity; this is followed by experimental validation with a representative system. Our workflow yielded size-excluded solvent and soluble porous organic cage pairs, leading to the realisation of a new porous liquid with enhanced methane uptake compared to previous systems discovered in a purely experimental high-throughput brute-force manner, highlighting the advantages of incorporating a computational workflow in the discovery of new porous liquids.

## Abstract 2

By combining solubility prediction software and a size-exclusivity prediction algorithm with a down-selection procedure and experimental validation steps, a computational workflow has been developed for the discovery of porous liquid solutions.

## Graphical abstract

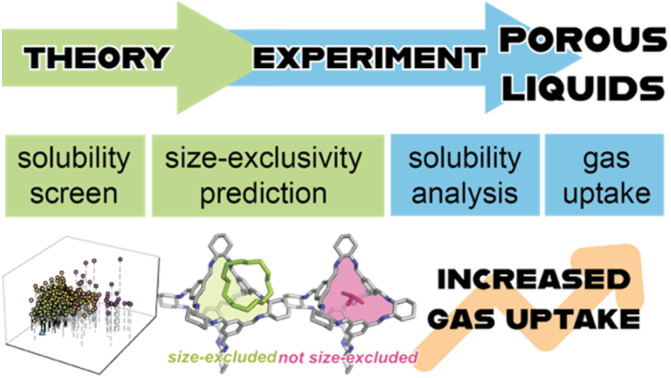

Porous liquids (PLs) are a relatively new sub-class of porous materials that combine the guest-accessible cavities of porous solids with the fast mass transfer of liquids, yielding a liquid featuring permanent ‘intrinsic’ porosity.^1,2^ This unique functionality was initially proposed by James and colleagues,^3^ leading to the categorization of PLs that was recently expanded into four types (Fig. 1a): type I – neat molecular liquids featuring permanent, intrinsic porosity; type II – empty molecular hosts dissolved in cavity-excluded solvents; type III – multiphase fluids featuring porous materials dispersed in cavity-excluded solvents; and later, type IV – neat, meltable extended porous solids.^4^ The recent experimental realisation of porous liquids has since motivated a host of potential applications to be investigated, including gas separation, gas storage, and catalysis,^5^ among others.^6^ Indeed, PLs have garnered attention as potential replacements for current industrial liquid sorbents,^7^ as they would be more easily implemented in existing industrial flow processes than their solid-state counterparts.

**Fig. 1:**
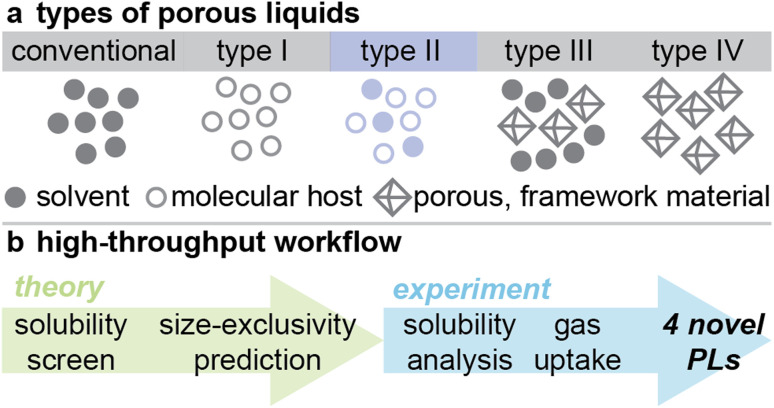
(a) Illustration of conventional liquids (featuring transient ‘extrinsic’ porosity) with the four types of PLs (featuring permanent ‘intrinsic’ porosity) – type I and IV PLs consist of neat porous hosts that are either in the liquid state or meltable respectively, whereas type II and type III consist of porous hosts either dissolved or dispersed in cavity-excluded solvents, respectively; (b) representative schematic summarising the computational workflow and experimental validation process presented in this work to streamline the discovery of type II porous liquid solutions.

Considering the diversity of potential applications and the component space, PLs are an interesting functional materials design problem. To select the ideal PL for a target application, an understanding of the macroscopic properties and the materials space that PLs span is required. Further, while each type offers intrinsic porosity, they differ in the ease of design, the required properties of their constituent components, and the overall properties of the resulting PL. For example, type I PLs possess higher viscosities compared to type II and III PLs and require materials exhibiting a low melting point without risk of decomposition or loss of porosity. Comparing type II and III PLs, both require cavity-excluded solvents, but the former relies on highly soluble porous material–solvent combinations, and the latter, being dispersions, require careful design to ensure stable dispersions are formed that do not suffer from phase separation (*i.e.*, creaming or sedimentation). The design rules for selecting a suitable solvent that both highly solubilizes the molecular host and is size-excluded are still unclear, and the move towards less toxic and lower cost solvents is also desirable for scale-up. Owing to these challenges, here we focus on the development of a workflow for type II PLs to streamline the identification of size-excluded and highly solubilising solvents, although the developed methods here would also be extensible to type III PLs where size-exclusivity and a poor solubilizing solvent is required.

Designing type II PLs for target applications may be considered as a general multi-objective optimisation problem; the ideal molecular host and solvent pair must be selected to generate a viable PL, ensuring the solvent is permanently cavity-excluded and that a reasonable pore concentration is achieved. At their initial discovery, one example of a type II PL was prepared based on a vertex-disordered imine porous organic cage (POC) mixture dissolved in a size-excluded perchlorinated solvent after a manual screen.^8^ Although seemingly simple, and while we have previously developed and reported an experimental high-throughput workflow to accelerate screening,^9,10^ this design problem is challenging in that it is simply too vast for brute-force experimental screening techniques. Indeed, there are estimated to be 10^60^ small molecules that could feasibly be synthesised; this does not account for the isoreticular and combinatorial materials that could be formed *via* the modular assembly of these molecular building blocks. Consider a modest database of 1000 molecular hosts and 10 000 candidate solvent molecules; this results in 10 million potential PLs, and is prohibitively large for exhaustive computation or experiment.

While computational studies may compliment experiment and offer atomistic insights into macroscopic properties, these measured properties are a result of interactions at varying time and length scales, and, thus, require models ranging from classical to quantum mechanical levels of theory.^7^ Indeed, while computational studies may require fewer resources than experiments, PL simulations are still time- and resource-intensive, often necessitating periodic models to assess bulk liquid properties and limiting the chemical space that can be feasibly explored using a high-throughput computational approach. This massive design space is further complicated by the youth of the PL field which limits data availability and, by extension, the applicability of data-driven methods, which typically require large amounts of data for predictive accuracy.

Chemical design initiatives for PLs have typically fallen into two main categories: (i) a brute-force screening approach – large-scale candidate testing based on resource availability or chemical intuition;^9,10^ or (ii) a down-selection or filtering approach – sequential constraints are placed on candidate combinations to decrease design space.^11^ Each of these approaches is limiting. For example, brute-force screening approaches like that reported previously for imine POC based type II PLs is resource- and time-intensive, requiring extensive synthesis and use of low accuracy measurements to increase throughput. Whereas high-performing candidates may be inadvertently excluded within a down-selection or filtering approach. Further, current computational methodologies employ several assumptions, which can limit their utility across PL formulations.^12^ Thus, alternative methods are required to assess the viability of potential pore carrier/solvent combinations.

Here, we present a computational, high-throughput workflow for identifying potential new PL solutions featuring two prediction algorithms to assess solubility and size-exclusivity of candidate pore carrier/solvent combinations. By combining solubility prediction software and a size-exclusivity prediction algorithm, and subsequently experimentally validating the predictions (Fig. 1b), we demonstrate the utility of these algorithms to design novel porous liquid solutions. While we employ a down-selection strategy for the purposes of experimental feasibility in this workflow demonstration, we emphasise the utility and efficiency of the predictive algorithms independently.

## Workflow and results

Our overall workflow for type II PL discovery involves a series of steps including: (i) solubility prediction and application of a series of selection parameters to identify potential highly solubilising solvents with desirable properties for a specific pore carrier; (ii) size-exclusivity prediction of the identified solvents from the porous motif using a custom algorithm that exploits information from atomistic simulations to identify potential pore carrier/solvent pairs that would successfully form a type II PL; followed by (iii) experimental validation *via* solubility and gas uptake measurements.

**Fig. 2:**
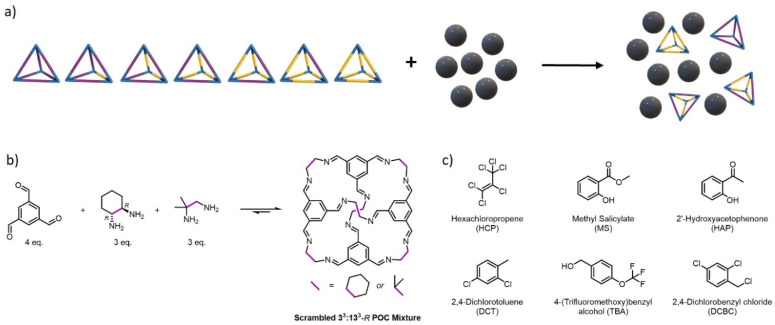
(a) Schematic illustrating the formation of a type II PL by dissolving a statistical distribution of ‘scrambled’ POCs in a size-excluded solvent (grey spheres); (b) synthesis of a mixture of vertex-disordered POCs *via* a dynamic imine condensation – combining 4 equivalents of 1,3,5-triformylbenzene with 3 equivalents of (1*R*,2*R*)-cyclohexane-1,2-diamine (used to form CC3) and 3 equivalents of 2-methylpropane-1,2-diamine (used to form CC13) affords a scrambled 3^3^**:**13^3^-*R* POC mixture; (c) the range of size-excluded solvents used previously to dissolve 3^3^**:**13^3^-*R* and form type II PLs.^9,10^

To develop and validate the proposed workflow, we first selected a representative pore carrier to serve as an initial case study. Of the candidate hosts for type II PLs, porous organic cages (POCs) present an example of an ideal molecular motif for PLs for several reasons: (i) POCs are discrete molecules containing a permanent cavity accessible through windows, and (ii) POCs possess an inherent solution processibility and maintain their pore structure upon dissolution. Owing to these factors, POCs have formed the basis of a substantial number of type II PLs to date.^13–15^ However, complicating the computational challenges, POCs generally possess fairly low solubilities overall. A number of design strategies have therefore been employed to increase their solubility enabling high concentration PLs to be realised, with the most popular being the formation of statistical ‘scrambled’ distributions of POCs with mixed vertex functionality to disrupt the solid-state packing and increase the solubility in a range of size-excluded solvents (Fig. 2a).^8–10^ In addition, one particular ‘scrambled’ POC, 3^3^**:**13^3^-*R* (Fig. 2b), has previously been screened in a wide range of solvents using both brute-force manual measurements and simulations, as well as high-throughput experimental screening,^9,10^ leading to a family of type II PLs by varying the size-excluded solvent (Fig. 2c), and also providing initial data for the solubility screening workflow here.

### Solubility candidate selection workflow

To access PLs with a reasonable pore volume capable of demonstrating an enhancement in gas uptake over the neat solvent, the pore carrier needs to be dissolved at relatively high concentrations (type II) or low concentrations to access high concentration dispersions (type III). For the former, solubility is typically determined using manual measurements in each solvent. Computationally assessing solubility is more challenging, owing to the complexity of competing inter- and intramolecular interactions. Previously, COSMO-RS, a continuum solvation model based on quantum chemical calculations, was used to estimate the solubility of one POC candidate with a series of solvents to design POC-based type II PLs.^11^ Yet, this method achieved only 58% accuracy, likely due to limitations associated with representing interactions between secondary and tertiary amines. Data-driven solubility prediction approaches have shown promise with respect to increased accuracy and robustness of the models.^16,17^ Yet, these models are trained on datasets of small organic molecules, which are not representative of the PL chemical space. While the candidate solvent molecules could be considered to fall within the small molecule datasets, the porous hosts would not – even the molecular hosts comprising type II PLs are too large for tools developed for small molecules. We therefore looked to develop an alternative, more accurate solubility assessment protocol for PLs.

Hansen solubility parameters (HSPs) provide a method to quantitatively assess the likelihood one molecular compound will dissolve another and have seen previous success across a variety of applications, ranging from pharmaceuticals, to polymers, and materials chemistry. For example, HSPs have been used to identify solvents that optimise the dispersion of nanomaterials such as graphene,^18^ to select green extraction solvents for bioactive compounds,^19^ to predict the dispersion of nanoparticles in polymeric films,^20^ to predict miscible mixtures for cocrystal formation of a drug and conformer,^21^ and to correlate the solubility parameters that govern self-assembly in molecular gels.^22^ Typically, however, HSPs are used to rationalise and predict the solubility behaviour of small molecules, polymers, and particles, rather than discrete large molecules such as candidate POCs. In addition, the requirement for solvent size-exclusivity to form type II PLs may affect the overall solubility and present a unique challenge. Therefore, here, we wanted to explore the applicability of HSPs to PL solubility prediction.

With HSPs, each molecule is associated with three polarity scales describing the energy density of dispersion forces (*δ*_d_), dipolar intermolecular forces (*δ*_p_), and hydrogen bonds (*δ*_h_). Solubility is then assessed by comparing the HSPs of the molecules of interest – the focal tenet being that molecules with similar HSPs are more likely to form a soluble mixture. To use HSPs in a predictive manner, we needed to determine the HSPs of the scrambled 3^3^**:**13^3^-*R* POC mixture; this is accomplished using the HSPiP software package.^23^ The solute parameters for the 3^3^**:**13^3^*-R* POC mixture are: *δ*_d_ = 22.3 MPa^½^ indicating a large dispersion contribution due to the large size of the cage molecules; *δ*_p_ = 0.1 MPa^½^ indicating there is no overall dipole across the cage molecule; and *δ*_h_ = 12.5 MPa^½^ indicating the cage molecule would form effective solutions with polar and hydrogen-bonding solutes. These parameters indicate that a non-polar solvent, with no permanent overall dipole but still polarisable, is preferential – this is represented by a high *δ*_d_ parameter and a low *δ*_p_ parameter. Halogenated solvents, and aromatic solvents to a lesser extent, provide this combination of polarity attributes, which agrees with the initial size-excluded solvents found to form highly concentrated PLs with 3^3^**:**13^3^-*R* (Fig. 2c).

Solubility of a candidate solute/solvent pair is then predicted by comparing the similarity of their HSPs. The Hansen interaction radius (*R*_a_) is quantified by calculating the distance between the HSPs of two substances in the three-dimensional Hansen space:

1 (*R*_a_)^2^ = 4(*δ*_d2_ − *δ*_d1_)^2^ + (*δ*_p2_ − *δ*_p1_)^2^ + (*δ*_h2_ − *δ*_h1_)^2^

Thus, molecules with lower *R*_a_ values and more similar HSPs are more likely to form a soluble mixture. Solubility prediction is achieved by experimentally determining the maximum suitable *R*_a_ value (*R*_0_). This ‘cut-off’ defines the radius of the resulting ‘solubility sphere’ centered on the solute. Here, all solvents within the sphere (and so *R*_a_ ≤ *R*_0_) are predicted to dissolve the solute.

**Fig. 3:**
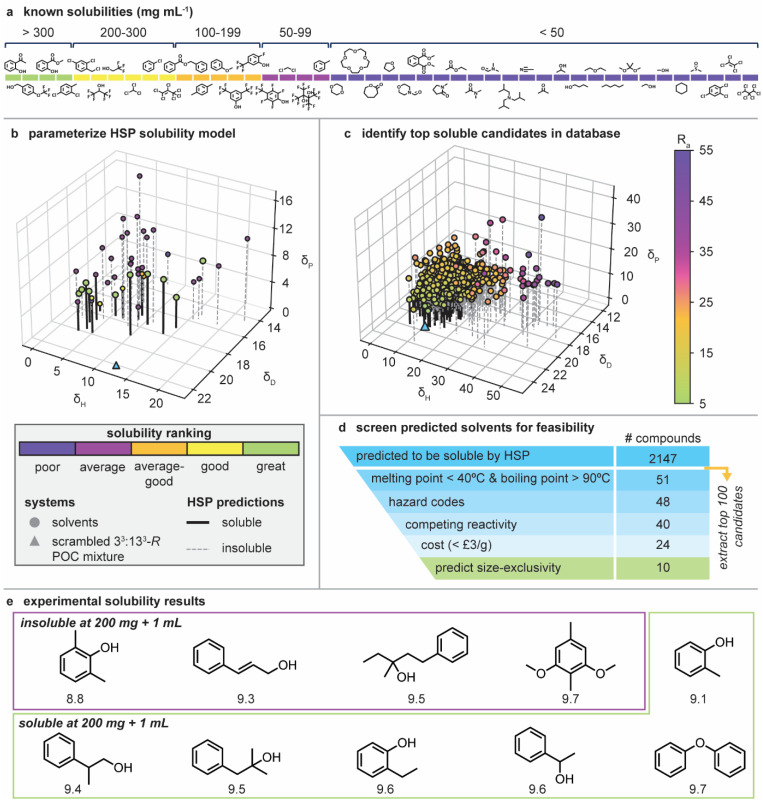
(a) Curated dataset of existing experimental solubility measurements of 3^3^**:**13^3^-*R* POC mixture in a range of solvents, including size-excluded examples; (b) the HSP model used to identify the solubility sphere of scrambled 3^3^**:**13^3^-*R* POC mixture (depicted as the blue triangle). Datapoints are colored based on their solubility ranking. Compounds predicted to form a solubilized mixture are highlighted by a bold, black line; (c) HSP comparison between the POC mixture (triangle data point) and the HSPiP database of candidate solvents (circle datapoints). Candidate solvent molecules are colored by their distance to the scrambled 3^3^**:**13^3^-*R* POC mixture (*R*_a_); (d) solvent down-selection procedure and associated number of candidate compounds remaining at each stage, with the top 10 from this down-selection procedure being selected and taken forward for experimental testing and size-exclusivity prediction; (e) top 10 selected solvents predicted to form a highly soluble mixture with the 3^3^**:**13^3^-*R* POC which meets all the selection criteria. These predictions were validated experimentally – four candidates were identified to be insoluble at the selected minimum 200 mg + 1 mL threshold (purple) and six candidates were identified to be soluble at 200 mg + 1 mL (green). Each candidate is labelled with its Hansen interaction radius.

To generate a predictive solubility model for the scrambled 3^3^**:**13^3^-*R* POC mixture, a dataset containing the previous solubility studies of the scrambled 3^3^**:**13^3^-*R* POC mixture in both conventional and size-excluded solvents was manually collated, and the resulting 44 solvents categorised based on the overall solubility of the POC (Fig. 3a and Table S1†).^9,10^ To ensure a reliable prediction method could be developed, the existing solubility data contained both poorly and highly solubilising molecules. This dataset was then used to predict the solubility sphere for the 3^3^**:**13^3^-*R* POC mixture using the HSPiP software (Fig. 3b). Using this solubility sphere, there was an 84% success rate on correlating the solvents with the solute parameters (Table S2†), *i.e.*, 84% of the solvents used to build the model would have been correctly predicted to solubilise the POC mixture. Furthermore, 86% of the known size-excluded solvents included in the model also correlated with the predictions. While a reasonably good success rate, there may be several reasons why this is not higher: (i) we are applying the model to a statistical distribution of a mixture of cage species instead of a single molecular species; (ii) the cage structure is quite complex, at least when compared to small molecules and polymers; and (iii) the dataset included size-excluded solvents, which do not solvate the entire cage molecule (*i.e.*, the cavity of the cage is unsolvated), although the prediction accuracy is quite consistent between the full solvent dataset and just the size-excluded solvent dataset, potentially suggesting that the solvation state of the cage cavity is a less contributing factor.

The approximately 10 000 compounds in the HSPiP database were subsequently ranked according to their calculated *R*_a_ (likelihood to solubilise the POC mixture), with 2147 potential new solvents identified (Fig. 3c). However, not all these candidates would be suitable as a PL solvent – in addition to needing to be size-excluded to ensure permanent porosity is maintained in the liquid state (addressed in the next stage of the workflow), it would also be beneficial for solvents to: (i) have a high boiling point to reduce volatility issues and to enable potential temperature-swing gas uptake/release cycles; (ii) have a relatively low melting point so that they are in the liquid state at, or near, room temperature; (iii) not have competing reactivity with the POC imine bonds due to their reversible nature; (iv) be less hazardous than some of those used previously (*i.e.*, avoid chlorinated solvents where possible); and (v) be affordable, which is especially important when considering scale-up. Thus, we implemented and applied a series of selection criteria as a screening procedure to the top 100 ranked solute compounds predicted by HSPiP (Fig. 3d and Table S3†). Starting with the closest match based on *R*_a_ and working progressively down the predicted solvents, the following selection criteria were therefore applied:

(a) Eliminate potential solvents that were not liquids at, or near, room temperature (melting point ≤ 40 °C), and that have a high boiling point (boiling point ≥ 90 °C), to ensure the selected solvents were liquids at reasonable temperatures and were comparable to previously reported PL solvents to avoid significant evaporation during experiments. These temperatures were selected so the solutions/mixtures could form liquids at room temperature (20 °C, assuming some melting point suppression could occur) and so solvent evaporation would not significantly affect the prepared samples when under experimental investigation.

(b) Eliminate solvents with functionality that could potentially react with the POC imines, such as amines and thiols, alongside any with significant hazards, including those fatal upon any exposure route, carcinogens, mutagens, or teratogens.

(c) Finally, cost was considered, and any solvents that were not commercially available at <£3/g were removed.^24^

Applying these criteria led to 24 solvents, of which, the top 10 solvents with an *R*_a_ below 9.7 MPa^½^ were taken forward for experimental validation of the solubility predictions and subsequent size-exclusivity screening (Fig. 3e).

### Experimental validation of solubility predictions

Prior to predicting the size-exclusivity of the potential solvents, we first validated the results of the solubility screening workflow by ensuring the scrambled 3^3^**:**13^3^-*R* POC mixture met a minimum solubility threshold of 200 mg + 1 mL. This concentration was selected as this was the solubility used in previous studies to compare PLs formed from the same scrambled POC mixture and corresponds to the ‘good’ solvent category in the HSPiP input set. See Sections S2 and S3† for a detailed description of synthetic and analytical methods.

Of the top 10 selected solvents, six were found to solubilise the POC mixture at the 200 mg + 1 mL threshold and remain liquid on dissolution (2-methylphenol, 2-ethylphenol, 1-phenylethanol, 2-methyl-1-phenyl-2-propanol, and 2-phenyl-1-propanol), one was found to solubilise the POC in a 200 mg + 1 mL sample at a slightly elevated temperature and remained liquid on cooling to room temperature (diphenyl ether – a low melting solid), one was found to solubilise the POC at 200 mg + 1 mL but solidified on cooling (2,6-dimethylphenol), and the remaining three solvents (3-phenyl-2-propen-1-ol, 1-phenyl-3-methyl-3-pentanol, 4-methyl-2,6-dimethoxyphenol) either did not fully dissolve the POC or underwent gelation (Table S4†). Overall, this led to a success rate of 70% when considering the solvents that solubilised the POC in a 200 mg + 1 mL sample. While this is slightly lower than the 84% accuracy of the HSP method when validated using previous experimental data (Table S1†), we believe this is a promising result, especially given we were only screening against a minimum threshold solubility over absolute solubilities. This promising results motivates future exploration of this solubility prediction method for porous liquid solutions. The six solvents that formed highly soluble mixtures and remained liquid at room temperature were taken forward for size-exclusivity prediction.

### Computational size-exclusivity prediction algorithm

As discussed above, ensuring the cavities of the pore carrier remain available for guest molecules is pivotal to PL performance. Pore availability is largely dictated by the relative sizes of the solvent, the cavity aperture, and the terminal chains of the pore carrier, which may interpenetrate cavities.^25^ While experimental efforts to assess size-exclusivity have evolved, they often infer size-exclusivity by measuring enhancements in gas uptake over the neat solvents,^7^ and experimentation is an infeasible solution to screening the vast chemical space spanned by PLs, even when considering a sub-set of these in the form of POC-based PLs like we are here. Computation offers a cost and resource effective alternative,^26^ and there are several computational methods for assessing size-exclusivity; these range in complexity from large-scale, atomistic molecular dynamics (MD) liquid simulations,^8,25^ to size comparison methods relying on static size measurements of pore carriers and solvent molecules. While effective, large-scale MD simulations are computationally intensive and prior studies have been limited to small (<10 models) datasets.^25^ Further, these methods require a new simulation for each pore carrier–solvent pair, and, as a result, is not feasibly implemented in potential, future HT workflows. While there are examples in the literature of size comparison methods (*i.e.*, comparing porous cavity diameters with solvent diameters),^11,12,27^ current methods do not account for the dynamics of each of the systems, and thus, struggle with accuracy and robustness. Therefore, we developed an alternative and efficient computational method for predicting size-exclusivity.

**Fig. 4:**
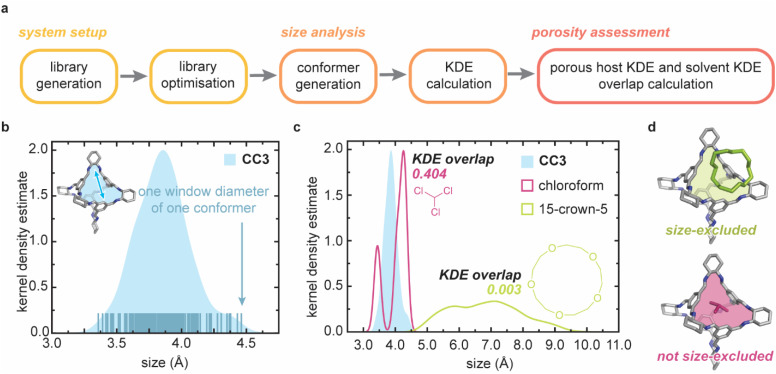
(a) The full workflow of the size-exclusivity prediction algorithm. A library of systems containing candidate porous hosts and solvents are prepared and optimised in separate workflows. Conformers are then extracted from a MD simulation, and the kernel density estimate (KDE) of sizes is calculated for each system. Finally, to predict the size-exclusivity, the KDE overlap is calculated for each porous host/solvent pair; (b) a sample KDE is presented for a representative POC, CC3. The KDE is derived from the distribution of cage apertures exhibited over the course of a MD simulation; (c) size exclusivity is quantified by the KDE overlap of the porous host/solvent pair. Two common solvents are presented; chloroform is not size excluded (KDE overlap > 0.35), while 15-crown-5 is size-excluded (KDE overlap < 0.25); (d) a schematic representation of the POC/15-crown-5 size-excluded result, and the POC/chloroform not size-excluded result.

The novel size-exclusivity prediction algorithm presented here is comprised of three main stages (Fig. 4a and S5†): (i) system setup; (ii) size analysis; and (iii) size-exclusivity assessment. POC and solvent molecules are treated independently until the final size comparison to determine size-exclusivity. Each stage is described briefly below using a representative and well-characterised POC, CC3.^28^ Full computational details of all stages are found in the ESI Section S5† and the code is available on Github (https://github.com/austin-mroz/SPLASHD).

#### System setup

First, POC and solvent single molecule structure models are generated and optimised using the software packages *stk* and *stko*,^29,30^ which support high-throughput structure generation and optimisation for supramolecular materials. To ensure that the computational dynamics are reasonable and align with experimental results, fifty conformers are then extracted from a MD simulation performed at the target experimental conditions (*i.e.*, temperature – 300 K); here, we examine standard temperature and pressure.

#### Size analysis

Second, for each of the fifty extracted conformers, the molecule size is assessed, and size is weighted by the energy of the conformer from which it was calculated. From these sizes, we generate a distribution using a kernel density estimate (KDE). Essentially, the KDE is a measure of the probability that the chemical system (POC or solvent) will take on a particular size and can be considered as a histogram of the dynamic dimensions of the system. In the case of PLs, we are concerned with measuring the pore carrier cavity window diameter, and the solvent dimensions. Thus, pore carriers and solvents are treated with separate size analysis workflows.

In the case of POCs, the window diameters are calculated using PyWindow^31^ for each window in each extracted conformer. These are then used to derive the KDE for the POC system (Fig. 4b). Using the distribution of pore apertures to predict guest selectivity has been previously used to identify POCs for xenon/krypton separations;^32^ the published cage window distribution is commensurate with the results we observe with our workflow.

In the case of solvent molecules, solvent dimensions are obtained using a custom size metric built on SMORES^33^ (Fig. S6†), an extension of conventional steric parameters, Sterimol.^34,35^ Here, we require a method that assesses all of the dimensions of the solvent molecule and, in doing so, the resulting distribution of solvent dimensions may be considered a measure of “shape”. Importantly, considering the complex solvent–cage interaction dynamics for candidate solvent molecules,^36^ we treat rigid and non-rigid candidate solvent molecules differently; the entire solvent molecule is considered for rigid candidates, whereas slices of the solvent molecules are considered for non-rigid candidates (Fig. S7†). Full details on calculating this metric are found in ESI Section S5.2.†Fig. 4c depicts the KDE for two candidate solvents, 15-crown-5 and chloroform, and further illustrates the unique characteristics of the custom size metric that we present. Here, chloroform is represented by a bimodal distribution, which indicates that the chloroform molecule shape is dominated by two main vectors – the Cl–Cl vector and the Cl–H vector. On the other extreme, 15-crown-5 is represented by a wide, unimodal distribution, reflecting the underlying symmetry of the molecule.

#### Size-exclusivity prediction

Lastly, size-exclusivity is quantified by the overlap of the porous host KDE and the solvent KDE; as a representative example, KDEs of two solvents (chloroform and 15-crown-5) and CC3 are compared, and the calculated overlaps are presented (Fig. 4c). We observe a large KDE overlap for chloroform/CC3, which indicates that the cage window and chloroform molecule are likely to take on conformations that are similar in size, and a small KDE overlap for 15-crown-5/CC3, which indicates that the 15-crown-5 solvent molecule takes on conformations that are larger than the cage window. This is expected considering chloroform is experimentally known to not be size-excluded, while 15-crown-5 is known to be size-excluded (Fig. 4d).^8,9^ The KDE overlap is used as a metric that assesses the probability that a cage window and solvent molecule will be similar sizes. Using the KDE overlap, we can classify cage/solvent pairs as porous (KDE overlap < 0.25), not porous (KDE overlap > 0.35), or potentially porous (0.25 < KDE overlap < 0.35). These designations are determined by the KDE kernel. Indeed, the KDE overlap cutoffs used to label candidate solvents (KDE overlap = 0.30) may be linked to the Gaussian kernel that was used to derive the KDE – within a standard Gaussian normal distribution, 34% of the area is one standard deviation away from the distribution mean. It should be noted that for the edge cases where the solvent molecule is sufficiently small and the pore carrier window distribution is sufficiently large enough to yield KDE overlap < 0.25, we further check the predictions by verifying that the mean of the pore carrier window distribution is smaller than the mean of the solvent size distribution. If this is not the case, the pore carrier/solvent pair is flagged for manual inspection.

**Fig. 5:**
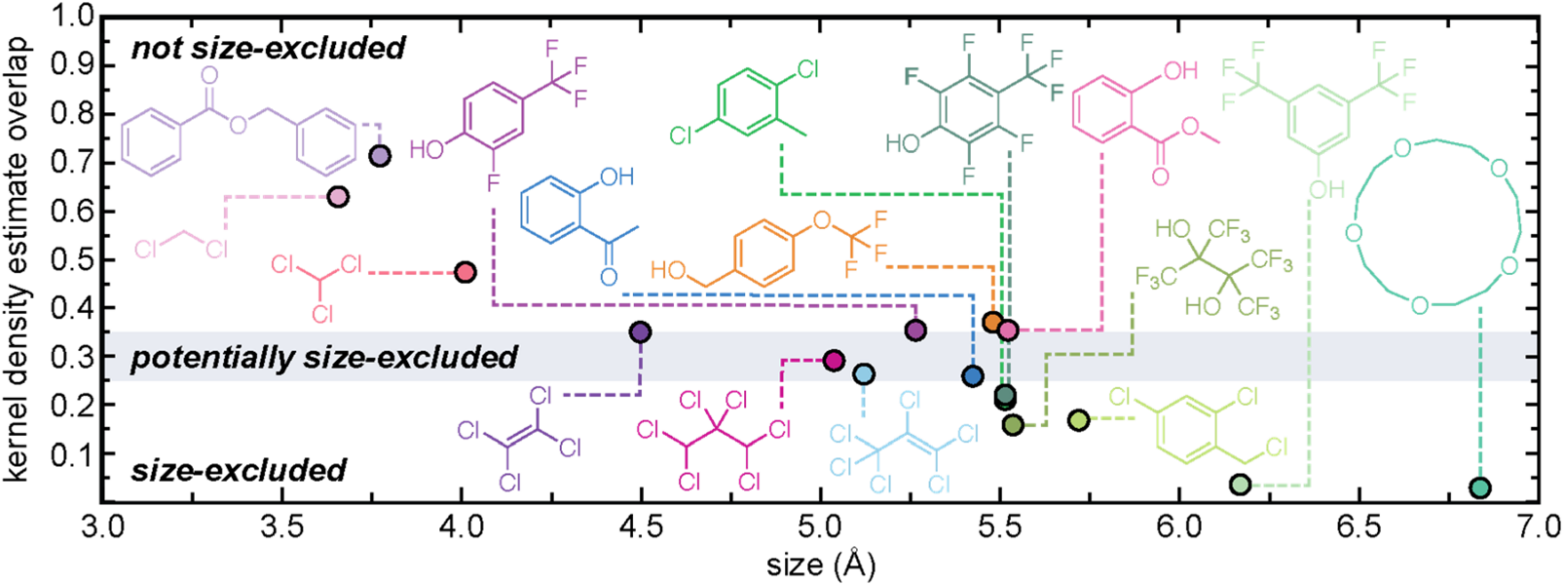
Previously reported solvents whose size-exclusivity from scrambled 3^3^**:**13^3^-*R* POC is known from experiment. Solvents are labelled as size-excluded (KDE overlap < 0.25), potentially size-excluded (0.25 < KDE overlap < 0.35), or not size-excluded (KDE overlap > 0.35), based on the calculated KDE overlap.

We validated our size-exclusivity prediction algorithm using a subset of the solvents from the curated database of solubility measurements above, focusing on those where it was experimentally known if the solvent was size-excluded from the cavities of the scrambled 3^3^**:**13^3^-*R* POC mixture or not (Fig. 5). We correctly predict the size-exclusivity of all the solvents in the validation set. The size-exclusivity algorithm suggested that four of the solvent molecules might be size-excluded; including 2′-hydroxyacetophenone, hexachloropropene, 1,1,2,2,3,3-hexachloropropane, and perchloroethylene. We can examine the KDE overlaps for each of the systems in relation to the experimental gas uptake results. Experimentally, 2′-hydroxyacetophenone (KDE overlap = 0.260) and hexachloropropene (KDE overlap = 0.264) are size-excluded. Whereas 1,1,2,2,3,3-hexachloropropane (KDE overlap = 0.292) and perchloroethylene (KDE overlap = 0.349) are not size-excluded. This result further reinforces the use of the KDE overlap as a size-exclusivity prediction metric; the lower the KDE overlap, the more likely the pore carrier/solvent are size-excluded, and *vice versa*.

**Fig. 6:**
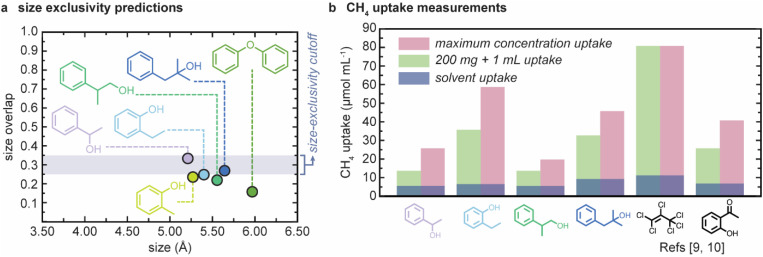
(a) Size-exclusivity predictions for solvents identified by the computational solubility screen. None of the six candidates are predicted to be not size-excluded; (b) calculated methane uptakes from ^1^H NMR spectra for the 4 new PL systems at 200 mg + 1 mL and the maximum concentration *vs.* the neat solvents, also compared to two previously reported high performing PL systems based on the same scrambled 3^3^**:**13^3^-*R* POC mixture at the same concentrations.^9,10^

The size-exclusivity of the candidate solvents identified by the computational solubility screening protocol were then predicted (Fig. 6a). From the calculated KDE overlaps, we see that diphenyl ether, 2-phenyl-1-propanol, 2-methylphenol, and 2-ethylphenol should be size-excluded, and 2-methyl-1-phenyl-2-propanol and 1-phenylethanol are potentially size-excluded. While four of the six solvents were predicted to be size-excluded, all combinations were taken forward as a means of validating the size-exclusivity prediction algorithm.

### Experimental validation of size-exclusivity *via* gas uptake enhancement measurements

The six POC/solvent pairs that met the solubility threshold (200 mg + 1 mL) above were screened for methane uptake using ^1^H NMR spectroscopy and compared to the neat solvents (Fig. 6b). Full experimental details are presented in ESI Section S6.† On addition of CH_4_ into the 2-methylphenol sample, the POC precipitated with the condensed CH_4_ behaving as an anti-solvent, meaning the uptake could not be determined. For the remaining five solutions, measured CH_4_ uptakes suggested that four new type II PLs had been formed with 2-ethylphenol, 2-phenyl-1-propanol, 1-phenylethanol, and 2-methyl-1-phenyl-2-propanol, which all showed significant uptake enhancement compared to the neat solvents, albeit to varying degrees (3.3 to 8.1 times, Fig. 6b). This success indicates the model is effective at size-exclusivity prediction. Furthermore, while CH_4_ uptake was recorded in the diphenyl ether cage solution (5.7 μmol mL^−1^), no direct comparison is made to its neat solvent counterpart as it forms a solid at ambient conditions, and therefore it cannot be determined whether a porous liquid has formed. Thus, we are also unable to use this solvent in the size-exclusivity validation.

The gas uptake in the new PLs based on 2-ethylphenol and 2-methyl-1-phenyl-2-propanol gave higher CH_4_ solubilities (36 μmol mL^−1^ and 33 μmol mL^−1^) than those discovered in the previous high-throughput experimental study at the same concentration (2′-hydroxyacetophenone, 26 μmol mL^−1^),^9^ though not meeting the uptakes recorded in the original scrambled PL based on the perchlorinated solvent discovered in 2017 (81 μmol mL^−1^).^10^ The CH_4_ uptake in the porous liquids based on 1-phenylethanol and 2-phenyl-1-propanol (both 14 μmol mL^−1^) were lower than 2-ethylphenol and 2-methyl-1-phenyl-2-propanol. The lower uptake for 1-phenylethanol reflects the prediction that this solvent is only potentially size-excluded based on the KDE, while the highest performing solvents were predicted to be excluded with more confidence. The downfield shift of the CH_4_ NMR signals (Table S8†) reflect the gas uptake enhancement, the values range from −0.97 ppm to −2.28 ppm in line with previously reported data, further indicating the CH_4_ molecules are occupying the cage pore as the electron rich arene walls shield the ^1^H nuclei from the external magnetic field.

As a final step, the maximum solubility of the four identified PLs was determined and the CH_4_ uptakes at the respective maximum solubility was measured (Fig. 6b). In previously published studies, the PLs were compared at the 200 mg + 1 mL formulation so the materials could be compared directly, without requiring density measurements.^9,10^ Gas uptake enhancement was shown to improve with the cage concentration previously. Similarly, here we observe enhanced CH_4_ uptake for the PLs at their maximum solubility for all samples (Fig. 6b). The chemical shift of the ^1^H environments in the CH_4_ gas are shifted further downfield in the NMR spectra (Table S8†), showing the increase in cavity concentration further moves the equilibrium towards gas occupying the pores present. The gas uptake in the 2-ethylphenol based PL at the maximum cage concentration gives a CH_4_ uptake capacity of 60 μmol mL^−1^. This exceeds the maximum uptake enhancement of the previously reported PL based on 2-hydroxyacetophenone (41 μmol mL^−1^) and reaches 74% of the uptake capacity of the PL based on perchloropropene, making this the highest performing non-chlorinated scrambled cage-based PL to date. It should be noted that in previous studies, additional purification of solvents was required to give the enhanced porosity recorded, here the high gas uptake and porosity was achieved using the as-purchased solvent. Again, the extent of porosity recorded in the PLs broadly correlates with the confidence at which size-exclusion was predicted, further validating the model. This also shows how experimentally, size-exclusion can be viewed as a continuous metric, with partial occlusion of the pore occurring with partially excluded solvents. While the model does not directly correlate with this, the KDE can reflect the level of porosity retained upon dissolution of the POC.

## Conclusions

We presented and experimentally validated a high-throughput computational workflow to identify potential candidate pore carrier/solvent PL combinations, as exemplified with type II POC-based PLs. At each step of the workflow, we identified several features contributing to the performance of the PLs designed using the scrambled 3^3^**:**13^3^-*R* POC mixture and demonstrated the applicability of these computational techniques to POC-based type II PL design.

Our study is the first application of HSPs for POC solubility prediction, obtaining an accuracy of 84% using the scrambled 3^3^**:**13^3^-*R* mixture and known solubility data. This approach is powerful considering the massive chemical space spanned by candidate solvents for PLs. While not an explicitly *in silico* method (*i.e.*, the solubility sphere of the candidate POC must be calculated using experimental results), HSPs allow us to exploit the wealth of information contained within often sparse solubility datasets. Of the 10 candidate solvent molecules, 6 were soluble at the tested threshold concentration. This highlights one of the limitations of HSPs for PL solubility prediction – this method mainly classifies solvents as soluble or insoluble. This classification is largely determined by the concentration of the experimental solubility data that was used to calculate the solubility sphere and may not be representative of other target concentrations. However, despite its relatively simple approach to solubility modelling, Hansen solubility theory is more than adequate to conduct a rationalised solvent screening without the need to consider the underlying fundamental thermodynamic parameters, making it accessible and quick to implement.

With respect to the integration of the size-exclusivity prediction algorithm in this work, we observe a significant decrease in the resources necessary to access this material property computationally. Indeed, computational size-exclusivity predictions would otherwise require full liquid simulations. There are two main contributions associated with this algorithm: (i) the procedure used to obtain a quantitative representation of molecular shape, and (ii) a quantitative metric for size-exclusivity *via* the KDE overlap. The candidate solvent size analysis workflow indicates the importance of solvent rigidity in size-exclusivity prediction. While intuitive, the dynamic nature of PLs necessitates new ways of quantifying molecular shape that account for the numerous degrees of freedom within candidate solvent molecules. Conventional size metrics are too rigid, in that they typically distill molecular shape into a series of vectors that are likely too large considering the dynamic interactions in the liquid state. By integrating a measure of rigidity into our workflow, we avoid this and gain a better representation of candidate solvents that assesses the size they would be as they interact with the cavity of the pore carrier in PLs. Indeed, the solvent size analysis workflow and molecular shape metric has implications beyond PLs in systems where molecular shape is a governing feature in property prediction.

The predictive success of this algorithm indicates that PL size-exclusivity is largely determined by the probability that the pore carrier cavity apertures and the solvent molecule shape are compatible (*i.e.*, they spend a large portion of time in shapes/sizes that are similar and/or the solvent molecule is smaller than the cavity window). While intuitive, the successful application of the KDE overlap quantifies this and provides a generalisable, candidate-agnostic framework that is amenable to the many porous materials or liquid carriers that are yet to be developed or applied as porous liquids, including inorganic systems and those featuring different functionalities.

Through this work, we identify four novel PLs featuring a scrambled 3^3^**:**13^3^-*R* mixture and demonstrate the viability of our computational workflow. While we study single-solvent systems here, we expect the generalisability of our algorithms and workflow to be extensible to mixed solvent systems. Specifically, mixed solvent system solubility could also be predicted for more concentrated solutions – on the basis that mixed solvents normally dissolve block co-polymers better, and we are using a mixture of cages. This also may have implications in the measured diffusivity of gases and viscosity. We expect the generalisability of the prediction algorithms and theoretical-experimental workflow presented here to be advantageous for and extensible to the accelerated discovery of POC-based type III PLs, as well as PLs which incorporate other porous materials as the pore carrier. Indeed, expanding to these systems merely requires incorporating additional, already-established, computational methods for assessing cavity window diameter for framework materials.

To validate the new computational methods presented in this workflow (application of HSP to POCs and the size-exclusivity prediction algorithm), we setup this study as a screening approach. Not only does this minimise the search space that we are exploring experimentally, but it helps to ensure positive ‘hits’ in the final experimental validation. While screening approaches for chemical design are useful, the close integration of computation and experimentation in chemical design and discovery are advantageous and may be achieved *via* closed-loop discovery. Here, machine learning algorithms are used to suggest the next set of experiments to perform to most efficiently optimise the objective; these methods have been shown to perform exceedingly well in chemical design and optimisation studies.^37,38^ Thus, we envision the true utility of the predictive methods presented in this work to be their application in closed-loop discovery workflows where they may be used to assess particular areas of chemical space without the need to perform resource-intensive experiments or simulations.

## Data availability

The data supporting this article have been included as part of the ESI,† and the related code can be found at https://github.com/austin-mroz/SPLASHD.

## Author contributions

R. L. G., A. M. M. and K. E. J. conceived the project, with R. L. G. leading the experimental workflow and K. E. J. leading the computational modelling. J. S. carried out the solubility prediction using HSPiP, R. C. M. designed and carried out the solvent down-selection procedure, B. D. E. and R. L. G. carried out the solubility screening, A. M. M. developed the computational size-exclusivity prediction algorithm and carried out the size-exclusivity predictions, and B. D. E. synthesised the scrambled cage and carried out the gas uptake measurements. The paper was written by A. M. M., B. D. E., and R. L. G., with input from all authors.

## Conflicts of interest

The authors declare no conflict of interest.

## Supplementary Material

SC-OLF-D5SC01875G-s001
