# Supplementary material for: Predicting pore-carrier solubility and size-exclusivity towards the rational design of type II porous liquid solutions
Source: Chem Sci. 2025 May 21;16(26):11897–907. doi: 10.1039/d5sc01875g (PMC12142219; doi:10.1039/d5sc01875g)
Supplement: SC-016-D5SC01875G-s001 [file SC-016-D5SC01875G-s001.pdf]

Supplementary Information (SI) for Chemical Science.
This journal is © The Royal Society of Chemistry 2025

Supporting Information

Predicting Pore-Carrier Solubility and Size-Exclusivity Towards the Rational Design of Type II Porous Liquid Solutions

Austin M. Mroz,[a,b†*] *Benjamin D. Egleston,*[a†] *James Sherwood,*[c] *Ruby C. Morel,*[a,d] *Kim E. Jelfs*[a] *and Rebecca L. Greenaway*[a*]

[†] These authors contributed equally.

[a] Department of Chemistry, Molecular Sciences Research Hub, Imperial College London, White City Campus, 82 Wood Lane, W12 0BZ, UK.

[b] I-X Centre for AI in Science, Imperial College London, White City Campus, 84 Wood Lane, London W12 0BZ, UK.

[c] Green Chemistry Centre of Excellence, University of York, Heslington, York, Yorkshire, YO10 5DD, UK.

[d] Department of Chemistry and Materials Innovation Factory, University of Liverpool, 51 Oxford Street, Liverpool, L7 3NY, UK.

Email: a.mroz@imperial.ac.uk, r.greenaway@imperial.ac.uk

Section S1. Solubility prediction via HSP

Table S1: Curated dataset of existing experimental solubility measurements of $\mathbf{3^3}$:$\mathbf{13^3}$-*R* POC mixture in a range of solvents, including size-excluded examples that have been previously reported to successfully form porous liquid solutions (highlighted in pale green). Each solvent is ranked and scored according to its measured solubility.

Solvent	Smiles	Solubility (mg/mL)	Ranking	Score
Chloroform	ClC(Cl)Cl	261	very good	1
Chlorobenzene	Clc1ccccc1	255	very good	1
1,1,1,3,3,3-Hexafluoro-2-propanol	OC(C(F)(F)F)C(F)(F)F	269	very good	1
2,2,2-Trifluoroethanol	OCC(F)(F)F	264	very good	1
Hexachloropropene	Cl/C(Cl)=C(Cl)\C(Cl)(Cl)Cl	242	very good	1
2,4-Dichlorobenzyl chloride	ClCc1ccc(Cl)cc1Cl	297	very good	1
2,4-Dichlorotoluene	Cc1ccc(Cl)cc1Cl	305	very good	1
4-(Trifluoromethoxy)benzyl alcohol	OCc1ccc(OC(F)(F)F)cc1	400	very good	1
2'-Hydroxyacetophenone	CC(=O)c1ccccc1O	600	very good	1
Methyl salicylate	COC(=O)c1ccccc1O	310	very good	1
p-Xylene	Cc1ccc(C)cc1	137.5	good	2
Methoxybenzene	COc1ccccc1	135.5	good	2
Benzyl benzoate	O=C(OCc1ccccc1)c2ccccc2	160.5	good	2
2,3,5,6-Tetrafluoro-4-(trifluoromethyl)phenol	Oc1c(F)c(F)c(C(F)(F)F)c(F)c1F	98.7	average-good	3
3,5-Bis(trifluoromethyl) phenol	Oc1cc(C(F)(F)F)cc(C(F)(F)F)c1	104.3	average-good	3
2-Fluoro-5-(trifluoromethyl) phenol	Oc1ccc(C(F)(F)F)cc1F	101	average-good	3
Dichloromethane	ClCCl	88.7	average	4
Toluene	Cc1ccccc1	68.8	average	4
Hexafluoro-2,3-bis(trifluoro-methyl)butane-2,3-diol	OC(C(F)(F)F)(C(F)(F)F)C(O)(C(F)(F)F)C(F)(F)F	79.3	average	4
Hexane	CCCCCC	<50	poor	5
Methanol	CO	<50	poor	5
Ethanol	CCO	<50	poor	5
Isopropanol	CC(C)O	<50	poor	5
Butanol	CCCCO	<50	poor	5
1,4-Dioxane	C1COCCO1	<50	poor	5
Acetonitrile	CC#N	<50	poor	5
Tetrahydrofuran	C1CCOC1	<50	poor	5
Ethyl acetate	CCOC(C)=O	<50	poor	5
Diethyl ether	CCOCC	<50	poor	5
Dimethylformamide	CN(C)C=O	<50	poor	5
Dimethyl Sulfoxide	CS(C)=O	<50	poor	5
N,N-Dimethylacetamide	CC(=O)N(C)C	<50	poor	5
1-Methyl-2-pyrrolidinone	CN1CCCC1=O	<50	poor	5
Acetone	CC(C)=O	<50	poor	5
4-Formyl morpholine	O=CN1CCOCC1	<50	poor	5
2,2-Dimethoxypropane	COC(C)(C)OC	<50	poor	5

Cyclohexanone	O=C1CCCCC1	<50	poor	5
15-Crown-5	C1COCCOCCOCCOCCO1	<50	poor	5
1,2,4-Trichlorobenzene	Clc1ccc(Cl)c(Cl)c1	<50	poor	5
1,1,2,2,3,3-Hexachloropropane	ClC(Cl)C(Cl)(Cl)C(Cl)Cl	<50	poor	5
Perchloroethylene	Cl/C(Cl)=C(Cl)/Cl	<50	poor	5
Dimethyl phthalate	COC(=O)c1ccccc1C(=O)OC	<50	poor	5
Triisobutylamine	CC(C)CN(CC(C)C)CC(C)C	<50	poor	5
ε-Caprolactone	O=C1CCCCCO1	<50	poor	5

Table S2: Calculated HSPs (energy contributions of dispersion forces (δ_d), dipolar intermolecular forces (δ_p), and hydrogen bonds (δ_h)) for each of the experimentally measured solvents and their similarity (Ra) with the **CC3³:13³**-*R* mixture, using the HSPiP software. Solvents are listed in order of increasing R_a and those highlighted in pale green indicate those previously reported to be size-excluded from the scrambled **CC3³:13³**-*R* mixture. The prediction accuracy column indicates whether the built model would have correctly predicted that the solvents would have solubilised the POC mixture.

Solvent	δ_d /MPa½	δ_p /MPa½	δ_h /MPa½	R_a /MPa½	Predicted to be soluble?	Is the prediction accurate?
Benzyl benzoate	20.0	5.1	5.2	9.9	yes	TRUE
1,4-Dioxane*	17.5	1.8	9.0	10.3	yes	FALSE
1,2,4-Trichlorobenzene	20.2	4.2	3.2	10.9	yes	FALSE
2'-Hydroxyacetophenone	19.2	8.9	10.1	11.0	yes	TRUE
1,1,1,3,3,3-Hexafluoro-2-propanol	17.2	4.5	14.7	11.3	yes	TRUE
Methoxybenzene	17.8	4.4	6.9	11.4	yes	TRUE
Chloroform	17.8	3.1	5.7	11.6	yes	TRUE
Methyl salicylate	18.1	8.0	13.9	11.6	yes	TRUE
2,4-Dichlorobenzyl chloride	19.8	6.8	4.0	11.9	yes	TRUE
2,4-Dichlorotoluene	19.1	4.6	3.4	11.9	yes	TRUE
4-(Trifluoromethoxy)benzyl alcohol	17.7	7.5	10.8	11.9	yes	TRUE
2-Fluoro-5-(trifluoromethyl) phenol	17.0	6.2	8.9	12.7	yes	FALSE
p-Xylene	17.8	1.0	3.1	13.0	yes	TRUE
1,1,2,2,3,3-Hexachloropropane	18.7	6.7	3.9	13.0	yes	FALSE
Chlorobenzene	19.0	4.3	2.0	13.0	no	FALSE
Tetrahydrofuran	16.8	5.7	8.0	13.1	no	TRUE
15-Crown-5	17.5	8.0	7.8	13.2	no	TRUE
Toluene	18.0	1.4	2.0	13.6	no	TRUE
Dichloromethane	17.0	7.3	7.1	13.8	no	TRUE
Hexachloropropene	18.6	2.9	0.9	14.0	no	FALSE
Butanol	16.0	5.7	15.8	14.1	no	TRUE

2,3,5,6-Tetrafluoro-4-(trifluoromethyl)phenol	15.9	4.2	7.6	14.2	no	TRUE
3,5-Bis(trifluoromethyl) phenol	16.1	4.8	7.2	14.2	no	TRUE
Cyclohexanone	17.8	8.4	5.1	14.3	no	TRUE
Isopropanol	15.8	6.1	16.4	14.8	no	TRUE
Ethyl acetate	15.8	5.3	7.2	14.9	no	TRUE
Dimethyl phthalate	18.6	10.8	4.9	15.0	no	TRUE
1-Methyl-2-pyrrolidinone	18.0	12.3	7.2	15.8	no	TRUE
Perchloroethylene	18.3	5.7	0.0	15.8	no	TRUE
N,N-Dimethylacetamide	16.8	11.5	9.4	16.1	no	TRUE
4-Formyl morpholine	18.6	14	8.9	16.1	no	TRUE
2,2,2-Trifluoroethanol	15.4	8.3	16.4	16.5	no	FALSE
Dimethylformamide	17.4	13.7	11.3	16.8	no	TRUE
Ethanol	15.8	8.8	19.4	17.1	no	TRUE
Diethyl ether	14.5	2.9	4.6	17.6	no	TRUE
2,2-Dimethoxypropane	14.8	4.8	4.0	17.8	no	TRUE
Acetone	15.5	10.4	7.0	17.9	no	TRUE
ε-Caprolactone	18.0	15.0	7.4	17.9	no	TRUE
Dimethyl Sulfoxide	18.4	16.4	10.2	18.2	no	TRUE
Triisobutylamine	15.0	1.3	1.3	18.4	no	TRUE
Hexafluoro-2,3-bis(trifluoromethyl) butane-2,3-diol	13.3	4.7	7.9	19.1	no	TRUE
Hexane	14.9	0.0	0.0	19.3	no	TRUE
Methanol	14.7	12.3	22.3	21.8	no	TRUE
Acetonitrile	15.3	18.0	6.1	23.6	no	TRUE

*Net zero dipole HSP values used.

Table S3: Top 100 to top 10 highlighted table based on selection criteria. Supplier prices were found only after the solvent was confirmed as a compatible and low hazard candidate. Prices were acquired from Sigma-Aldrich (accessed Oct. 2022).

R_a	Name	Melting Point (°C)	Boiling Point (°C)	Containing Mutagenic, Carcinogenic, Teratogenic or Fatal Hazards	Competing Reactivity	Price per Gram
5.7	2-naphthalenthiol	81	286	N	Y	N/A
5.9	2-phenyl-*p*-phenylenediamine	75	354	N	Y	N/A
6.0	2,4-diaminodiphenylamine	131	-	Y	Y	N/A
6.4	4,4'-diaminodiphenylamine	300	-	N	Y	N/A
6.7	benzenethiol	-15	169	Y	Y	N/A
6.7	1-naphthol	96	288	N	N	N/A
7.2	toluene-3,4-diamine	89	156	N	Y	N/A
7.9	2,6-dimethylthiophenol	-	122	N	Y	N/A
8.2	4-methylbenzenethiol	41	195	N	Y	N/A
8.2	2-thienyl mercaptan	-	-	N	Y	N/A
8.3	Diphenolic acid	171.5	-	N	N	N/A
8.4	2-phenylenediamine	103	257	Y	Y	N/A
8.6	2-methyl-1-naphthol	64	-	N	N	N/A
8.7	*N,N*-dimethyl-*p*-phenylenediamine	36	262	Y	Y	N/A
8.8	2,6-xylenol	45	203	N	N	N/A
8.8	2,5-xylenol	78	212	N	N	N/A
8.8	3,4-xylenol	68	227	N	N	N/A
8.9	2-ethylthiophenol	-	203	N	Y	N/A
8.9	2-mercaptoanisole	-	99	N	Y	N/A
9.0	2-methylaminophenol	89	-	N	Y	N/A
9.0	3,4-methylenedioxyaniline	41	144	-	Y	N/A
9.0	Phenylacetaldehyde glyceryl acetal	-	-	N	N	Perfume additive*
9.1	*o*-cresol	29	191	N	N	£1.56
9.1	phenylacetic acid	77	265	N	N	N/A
9.2	2,3,6-trimethylphenol	58	226	N	N	N/A
9.2	Benzoic acid	125	133	N	N	N/A
9.3	Cinnamyl alcohol	33	250	N	N	£0.05
9.3	2-methoxynaphthalene	70	274	N	N	N/A
9.3	2-furfurylpyrrole	-	78	N	N	N/A
9.3	2-methylthiophenol	150	195	N	Y	N/A
9.4	Cinnamic acid	136	300	N	N	N/A

9.4	β-methylphenethyl alcohol	-37	111	-	N	£0.52
9.4	Phenol	42	182	Y	N	N/A
9.5	2-amino-*m*-cresol	149	240	N	Y	N/A
9.4	Vanillin propylene glycol acetal	-	-	-	N	Unavailab le
9.4	*p*-α-trimethylbenzyl alcohol	-	64	-	N	N/A
9.5	1-phenyl-3-methyl-3-pentanol	-	112	N	N	£1.58
9.5	2-methyl-1-phenyl-2-propanol	23	94	N	N	£0.13
9.5	DL-phenylalanine	318	-	-	Y	N/A
9.5	Phenylalanine	270	280	N	Y	N/A
9.6	naphthalen-2-yl-2-aminobenzoate	118	-	N	Y	N/A
9.6	α-methylbenzyl alcohol	19	204	N	N	£0.80
9.6	2-methoxybenzoic acid	98	-	N	N	N/A
9.6	2-thienyl disulfide	56	-	N	Y	N/A
9.6	2-methyl-3-furanthiol	-	57	Y	Y	N/A
9.6	2-ethoxy-4-(4-methyl-1,3-dioxolan-2-yl)phenol	-	-	-	N	Perfume additive*
9.6	3-nitro-*o*-toluidine	92	305	N	Y	N/A
9.6	2-ethylphenol	-18	195	N	N	£1.84
9.7	Vanillin-1,2-butylene glycol acetal	-	-	-	N	Perfume additive*
9.7	1-phenylpropionic acid	136	-	N	N	N/A
9.7	4-methyl-2,6-dimethoxyphenol	37	145	N	N	£1.20
9.7	diphenyl ether	25	259	N	N	£0.24
9.7	1-methoxy-4-methylphenol	-32	175.5	Y	N	N/A
9.7	*Cis*-anol	-	229.6	-	N	Perfume additive*
9.7	Phenoxyacetic acid	98	285	N	N	N/A
9.7	1-(*p*-tolyl)ethanol	-	218	-	N	£1.89
9.8	Resorcinol acetate	-	283	N	N	£0.23
9.8	3-methyl-1-phenylbutan-2-ol	-	-	N	N	Perfume additive*
9.8	Anisyl phenylacetate	-	370	-	N	£1.32
9.9	Isoeugenol	27	132	N	N	£0.20
9.9	2-vinylphenol	-	-	N	N	Perfume additive*
9.9	4-ethylguaiacol	15	234	N	N	£0.80
9.9	4-ethyl-2,6-dimethoxyphenol	-	-	-	N	Perfume additive*

10.0	*m*-cresol	8	203	N	N	£0.57
10.0	2-phenyl-1-propanol	-	219	N	N	£0.36
10.0	2-allylphenol	-6	220	N	N	£0.20
10.0	4-propenyl-2,6-dimethoxyphenol	-	-	-	N	Perfume additive*
10.0	Guaiacyl phenylacetate	32	-	-	N	£0.70
10.0	Benzophenone-7	96	-	N	N	N/A
10.0	Deoxycholic acid	177	-	N	N	N/A
10.0	1-phenyl-3-buten-2-ol	-	-	N	N	£8.52
10.0	Tyramine	160	175	N	Y	N/A
10.0	β-naphthyl ethyl ether	37.5	282	N	N	£0.49
10.1	Anisyl alcohol	22	259	N	N	£0.18
10.1	2-methoxy-4-vinylphenol	57	224	N	N	N/A
10.1	2,5-dimethyl-3-furanthiol	-	-	N	Y	N/A
10.1	Isoeugenyl phenylacetate	-	-	-	N	Perfume additive*
10.1	4-allyl-2,6-dimethoxyphenol	-	168	N	N	£8.00
10.1	benzophenone-3	66	155	N	N	N/A
10.1	2,2'-(dithiodimethylene) difuran	10	112	-	Y	N/A
10.1	1-phenyl-2-butanol	-	124	N	N	£2.30
10.2	Propenyl guaethol	86	-	N	N	N/A
10.1	Isoeugenyl benzyl ether	58	-	-	N	N/A
10.2	Bis(2-Methyl-3-Furyl) tetrasulfide	-	-	N	Y	N/A
10.2	Benzyl phenylacetate	-	317	-	N	£4.34
10.2	2-isopropylbenzyl alcohol	-	-	-	N	Unavailab le
10.2	2,4-difurfurylfuran	-	-	-	N	Unavailab le
10.2	Cholic acid	198	-	N	N	N/A
10.2	Phenyl disulfide	58	310	N	Y	N/A
10.2	2-methoxy-4-propylphenol	-	125	N	N	£0.55
10.3	2-propylphenol	7	220	N	N	£1.75
10.3	Furfuryl 2-methyl-3-furyl disulfide	-	280	N	Y	N/A
10.3	4-propyl-2,6-dimethoxyphenol	-	-	-	N	£8.24
10.3	*p*-tolyl phenylacetate	76	-	-	N	N/A
10.4	1,2,4-benzenetriacetate	-	-	-	N	£1.60

10.4	4-methoxybenzoic acid	185	276.5	N	N	N/A
10.4	B-caryophyllene alcohol	94	-	N	N	N/A
10.4	2-nitro-*m*-phenylenediamine	-	-		Y	N/A
10.4	Cinnamyl cinnamate	42	370	-	N	£1.69
10.4	2-nitro-*p*-phenylenediamine	140	-	N	Y	N/A

* **Perfume additives** were available through perfumery wholesalers at high cost; these were eliminated.

Section S2. General synthetic and analytical methods

Materials: All reagents were used as received unless otherwise stated. 1,3,5-Triformylbenzene was purchased from Manchester Organics; (1*R*,2*R*)-cyclohexane-1,2-diamine, 1,2-diamino-2-methylpropane and 1-phenyl-3-methyl-3-pentanol (>98%) were purchased from TCI UK. Cinnamyl alcohol (98% trans) and *o*-cresol (99% AcroSeal®) were purchased from Acros Organics. 4-Methyl-2,6-dimethoxyphenol (97%) and (±)-2-phenyl-1-propanol (97%) were purchased from Alfa Aesar. Diphenyl ether (≥99.9%) was purchased from Supelco. 2,6-Dimethylphenol (99%), 2-ethylphenol (99%), 1-phenylethanol (98%) and 2-methyl-1-phenyl-2-propanol (98%) were purchased from Aldrich. Dichloromethane (DCM) and ethyl acetate were HPLC grade and purchased from VWR. Chloroform-*d* was purchased from Fluorochem (UK) and was stored over activated 3Å molecular sieves and sodium carbonate. All other reagents were purchased from Sigma Aldrich.**Synthesis:** All reactions were stirred magnetically using Teflon-coated stirring bars. Removal of solvents was done using a rotary evaporator.

Nuclear Magnetic Resonance (NMR): ^{1}H Nuclear magnetic resonance (NMR) spectra were recorded using an internal deuterium lock for the residual protons in $CDCl_3$ (δ = 7.26 ppm) or CD_2Cl_2 (δ = 5.32 ppm) at ambient probe temperature on a Bruker Avance 400 (400 MHz) spectrometer. NMR studies to calculate the CH_4 uptake were conducted using in-house made calibrated capillary of TMS in CD_2Cl_2, so as to have no effect on the studies of the porous liquid. NMR data are presented as follows: chemical shift, integration, peak multiplicity (s = singlet, d = doublet, t = triplet, q = quartet, m = multiplet, br = broad, app = apparent) and coupling constants (*J* / Hz). Chemical shifts are expressed in ppm on a δ scale relative to δ_{CDCl3} (7.26 ppm) and coupling constants, *J*, are given in Hz.

HRMS: High resolution mass spectrometry (HRMS) was carried out using an Agilent 6545XT AdvanceBio LC/Q-TOF ESI mass spectrometer (capillary voltage 4000 V, fragmentor 225 V) in positive-ion detection mode. The mobile phase was LCMS grade MeOH containing 0.1% formic acid at a flow rate of 0.25 mL/min.

Section S3. Synthesis of scrambled 3^3:13^3-*R* POC mixture

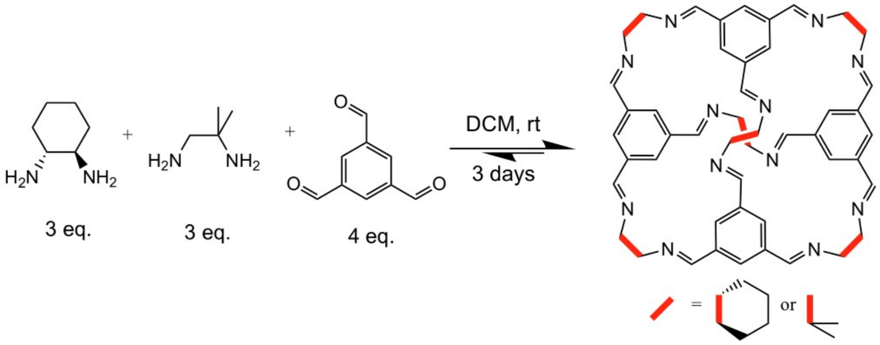

To a solution of 1,3,5-triformylbenzene (6.20 g, 38.2 mmol, 4.0 equiv.) in DCM (800 mL), was added solutions of (1*R*,2*R*)-cyclohexane-1,2-diamine (3.27 g, 28.6 mmol, 3.0 equiv.) in DCM (150 mL), and 1,2-diamino-2-methylpropane (2.53 g, 28.7 mmol, 3.0 equiv.) in DCM (150 mL). The resulting reaction mixtures were stirred at room temperature until completion, as analysed by ^{1}H NMR spectroscopic analysis (3 days). The solution was concentrated *in vacuo*, and the resulting crude cream solid was washed with ethyl acetate (3 × 100 mL), collected by filtration, dried under vacuum at ambient temperature, and then desolvated in a vacuum oven at 90 °C overnight, affording an off-white solid which was used for the subsequent porous liquid studies (4.87 g, 49%).

^{1}H NMR (400 MHz, $CDCl_3$) δ_H (ppm) 8.20-7.83 (24H, m, N=CH and ArH), 3.95-3.37 (12H, m, CHN=CH), 1.85-1.34 (42H, m, CH_2 and CH_3); **^{13}C NMR** (101 MHz, CDCl3) δ_C (ppm) 161.0, 159.1, 155.3, 137.1, 136.6, 129.4, 74.7, 72.3, 61.2, 33.1, 29.5, 24.4, 22.1; **HRMS** (ES+) calc. for scrambled cages **$3^0$13^6** $C_{60}H_{72}N_{12}$ 960.6003, **$3^1$13^5** $C_{62}H_{74}N_{12}$ 986.6159, **$3^2$13^4** $C_{64}H_{76}N_{12}$ 1012.6316, **$3^3$13^3** $C_{66}H_{78}N_{12}$ 1038.6472, **$3^4$13^2** $C_{68}H_{80}N_{12}$ 1064.6629, **$3^5$13^1** $C_{70}H_{82}N_{12}$ 1090.6785, **$3^6$13^0** $C_{72}H_{84}N_{12}$ 1116.6942; found $[M+H]^+$,961.6179, 987.6303, 1013.6465, 1039.6622, 1065.6771, 1091.6922, and $[M+2H]^{2+}$ 483.5155, 494.3302, 507.3313, 520.3394, 533.3485, 559.3549. Data in accordance with literature values.[1]

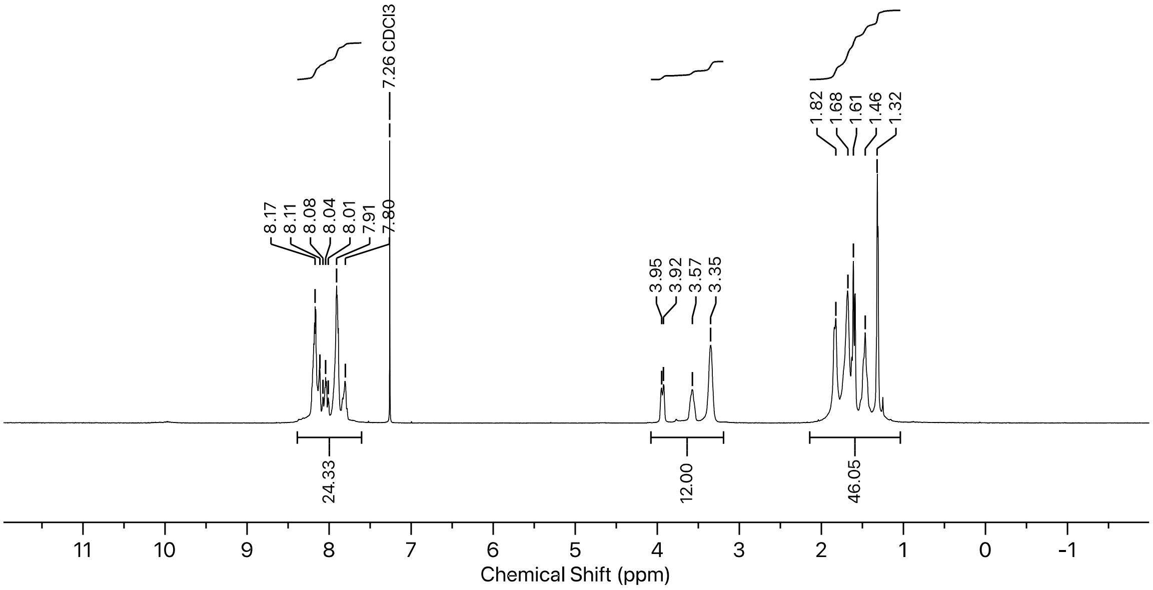

Figure S1: ^{1}H NMR ($CDCl_3$) spectrum of the scrambled 3^3:13^3-*R* POC mixture.

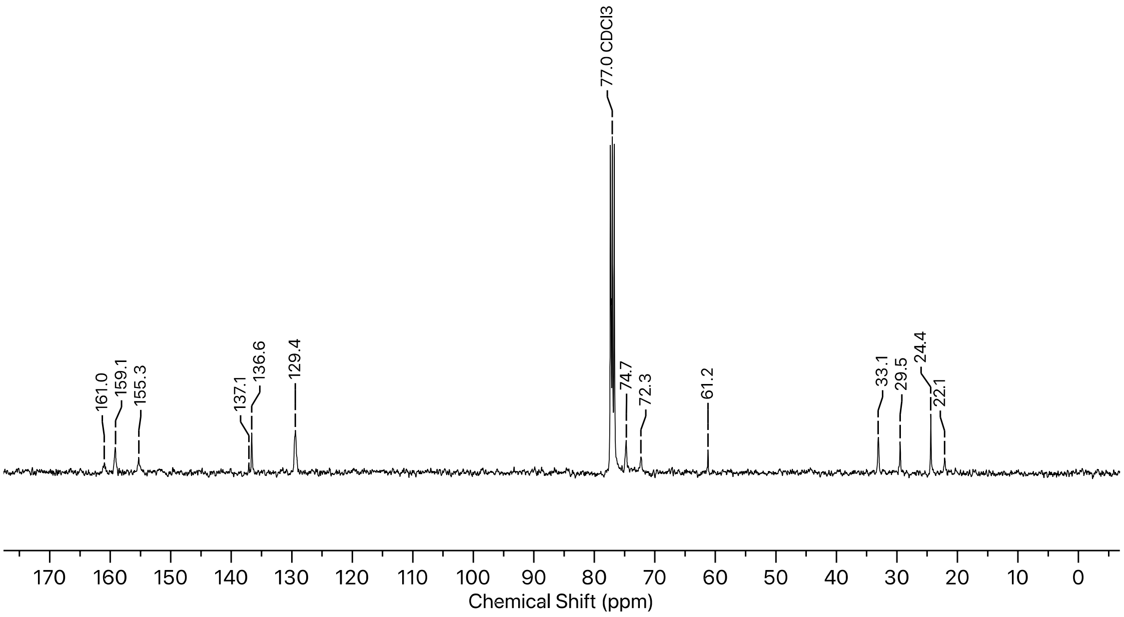

Figure S2: ^{13}C NMR ($CDCl_3$) spectrum of the scrambled **3^3:13^3**-*R* POC mixture.

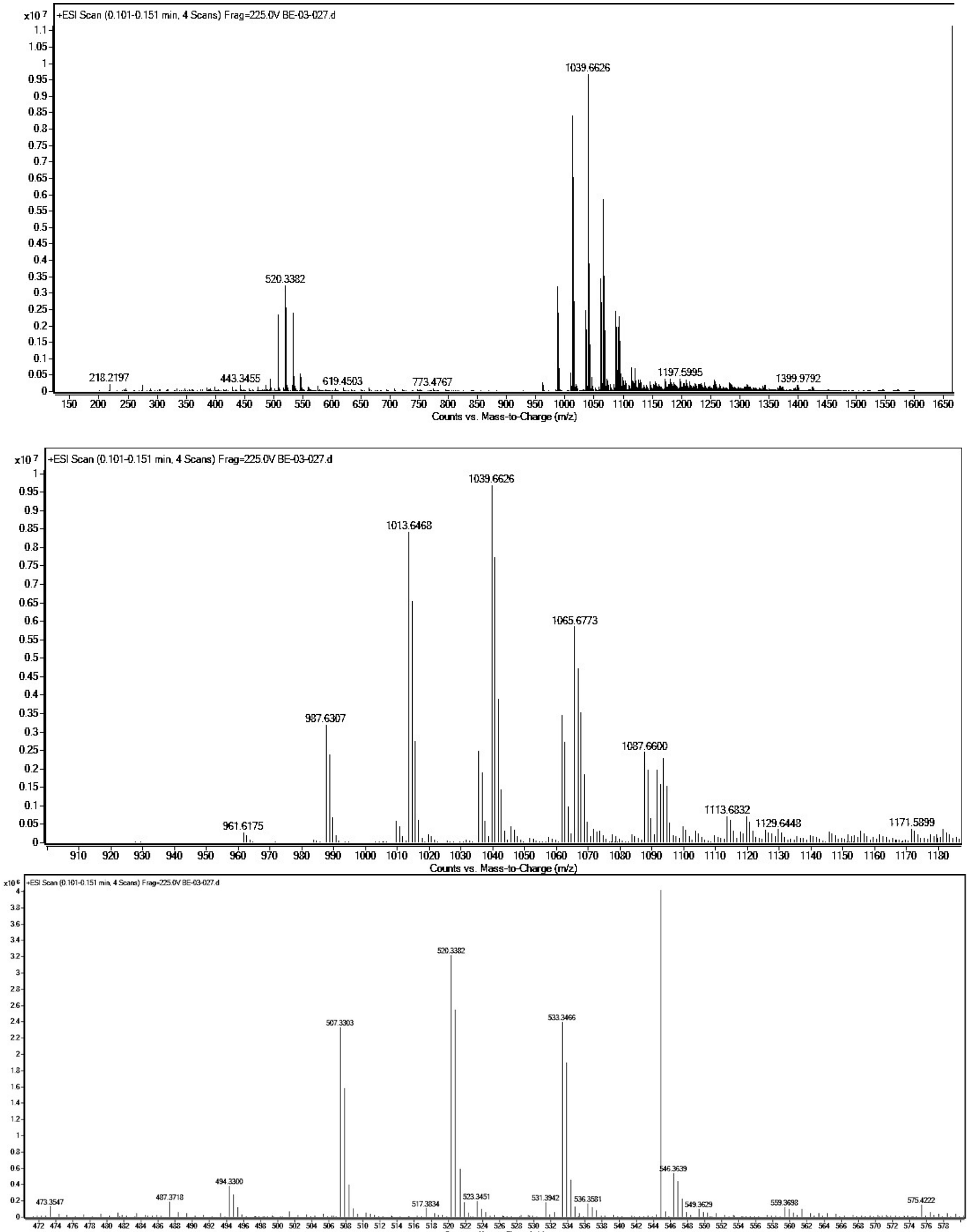

Figure S3: HRMS spectrum of the scrambled **3^3:13^3**-*R* POC mixture (top), with expansions of the $[M+H]^+$ peaks (middle) and $[M+2H]^{2+}$ peaks (bottom).

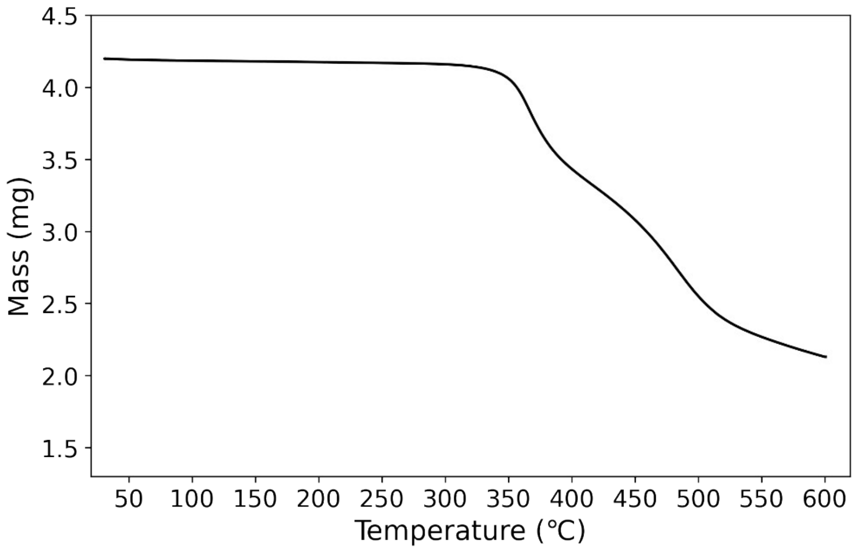

Figure S4: Thermogravimetric analysis of the $\mathbf{3^3{:}13^3}$-*R* POC mixture.

Section S4. Experimental Solubility Measurements

Highly Soluble Combinations Screen: An initial solubility test was carried out on the top 10 selected candidates based on a threshold concentration to be selected for gas uptake experiments – 1 mL of the selected solvent was added to 200 mg of scrambled $\mathbf{3^3{:}13^3}$-*R* cage in a vial fitted with a stirrer bar and the samples were stirred at room temperature for 2 hours before the vial contents were inspected for dissolution. The observations of this experiment are summarised in **Table S4**.

Table S4: Solubility of scrambled $\mathbf{3^3{:}13^3}$-*R* cage screened in top 10 solvents from HSPiP software – 10 x 200 mg scrambled cage samples weighed into vials, and 1 mL of each solvent added to each vial. Samples were stirred and sonicated before being inspected to determine if fully dissolved. If solvent was low melting, sample was gently heated on a hotplate with stirring until sample dissolved before allowing to cool to room temperature; in these cases, melting point (mpt) is indicated.

Reagent	Molecular Weight	Density (g/mL)	Scrambled Cage fully dissolved at 200 mg + 1 mL?	Carried forward for gas uptake?
1-phenyl-3-methyl-3-pentanol	178.27	0.938 (25 ^{o}C)	No - and formed a gel	✕
4-methyl-2,6-dimethoxyphenol	168.19	(mpt 37-42 ^{o}C)	Partially dissolved the cage, and remained liquid on cooling	✕
Cinnamyl alcohol/3-phenyl-2-propen-1-ol	134.18	1.044 (25 ^{o}C)	No - solidified	✕
2,6-dimethylphenol	122.16	(mpt 43-45 ^{o}C)	Yes - briefly heated to melt solid and then stirred at room temp, resolidified when cooled so was not carried forward for gas uptake	✕
o-Cresol/2-methylphenol	108.14	1.05 (20 ^{o}C)	Yes - stirred at room temp for a couple hours, still liquid stirring O/N, not too viscous	✓
2-ethylphenol	122.16	1.037 (25 ^{o}C)	Yes - stirred at room temp for a couple hours, still liquid stirring O/N, not too viscous	✓
1-phenylethanol	122.16	1.012 (25 ^{o}C)	Yes - stirred at room temp for a couple hours, still liquid stirring O/N, not too viscous	✓
2-methyl-1-phenyl-2-propanol	150.22	0.974 (25 ^{o}C) (mpt 23-25 ^{o}C)	Yes - briefly heated to melt solid and then stirred at room temp, no re-solidification	✓

			observed and still liquid after stirring O/N, fairly viscous	
2-Phenyl-1-propanol	136.19	0.975 (25 ^{o}C)	Yes, stirred at RT O/N, still liquid but quite viscous	✓
Diphenyl ether	170.21	(mpt 25-27 ^{o}C)	Yes - briefly heated to melt solid and then stirred at room temp, no re-solidification observed, still liquid stirring O/N, not too viscous	✓

Maximum Solubility Measurement: Of the 5 candidate solvents after threshold solubility screening and initial gas uptake measurements, 4 were then taken forwards for maximum solubility measurements to improve CH_4 uptake capacity through increased pore loading – *o*-cresol was eliminated due to precipitation of the scrambled cage upon exposure to CH_4. The solubility was determined by adding a small amount of scrambled cage (Table S6) to a tared glass vial followed by incremental addition of the selected solvent until full dissolution was observed. The total mass of the sample was record from which the maximum cage loading could be calculated as a percentage of the weight (**Table S5**).

Table S5: Recorded maximum solubility of scrambled **CC3^3:13^3**-*R* mixture in each of the final 4 solvents.

Solvent	Scrambled cage mass (mg)	Solvent Mass (mg)	Concentration
1-Phenylethanol	189.9	727.2	20.9% w/w
2-Ethylphenol	128.8	517.6	19.9% w/w
2-Phenyl-1-propanol	126.2	574.9	19.9% w/w
2-Methyl-1-phenyl-2-propanol	116.3	529.8	17.8% w/w

Section S5. Computational size-exclusivity

The size exclusivity algorithm presented in this work is comprised of three main stages, ***Figure S5***: 1) system setup, 2) size analysis, 3) size exclusivity assessment. Here, we describe each stage in detail.

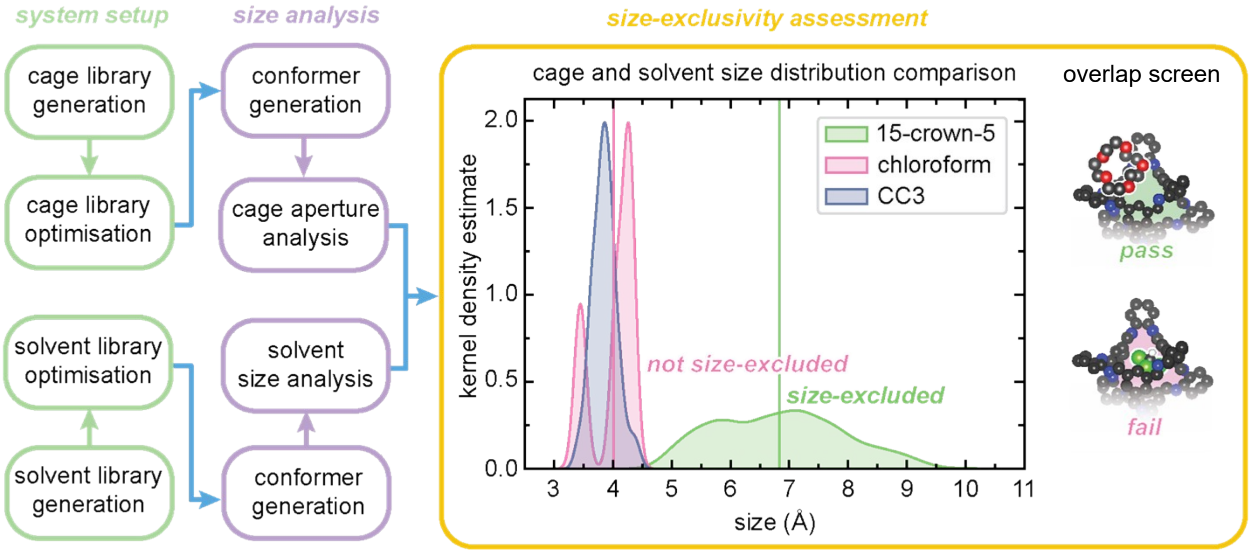

Figure S5: The full workflow of the computational size-exclusivity algorithm. This is comprised of three main stages: (1) system setup; (2) size analysis; and (3) size exclusivity assessment.

Section S5.1 System setup

Cage and solvent molecule models are generated using the *stk* software package[2]; *stk* facilitates the construction of geometric models for several materials classes, including supramolecular materials, covalent-organic frameworks, polymers and rotaxanes. Structures are optimized using OPLS3[3] forcefield from MacroModel[4,5] and facilitated by the *stko*[6] software package. A gas phase molecular dynamics (MD) simulation is performed at desired experimental conditions, ***Table S6***. 50 conformers are extracted from the MD trajectory as a *.xyz* file for size analysis (***Section S5.2***).

Table S6: The molecular dynamics simulation parameters.

Temperature (K)	300
Time step (fs)	0.7
Equilibration time (ps)	50
Simulation time (ps)	10000
Minimum gradient (kJ/Å-mol)	1.0

Notably, this methodology negates the effect of solvent on the cavity window diameters of the pore carriers. Indeed, previous studies have shown that solvent affects the pore window diameter for porous organic cage-based porous liquids. For example, we have previously shown for porous ionic liquids that the presence of solvent results in a narrower cage cavity diameter distribution,[7] and Rimsza *et al.* showed computationally that porous organic cage solvation results in a variation in the cage window diameter.[8] However, accounting for solvent effects at this step of the size-exclusivity prediction algorithm would limit the ability of the methodology to extrapolate – requiring multiple simulations for the same system and, subsequently, requiring more computational resources and time. Thus, to minimise computational

resources, we prioritise a solvent-agnostic approach – and assume that the effect of solvent is sufficiently negligible and the error associated with this approximation is accounted for in the lenient size-exclusion size-exclusivity classification where solvents may be labeled as "potentially size-excluded" (Section S5.3).

Section S5.2 Size analysis

Considering we are examining proxy host-guest systems, we are naturally concerned with quantifying the void space of the cage and the occupied space of the candidate solvent molecules. Specifically, we quantify the cage window diameters and the solvent dimensions since size-exclusivity is determined by whether the solvent can travel through the cage window. Thus, the size assessment protocols for these systems are different.

Cage window analysis – PyWindow[9] is used to quantify the cage window diameters for each extracted cage conformer.

Solvent dimension analysis – We quantify the solvent dimensions according to a custom metric and workflow.
Custom size metric – The custom size metric developed for this workflow is based on the conventional Sterimol parameters, which were originally presented in 1976 by Verloop for drug discovery[10] and recently applied for asymmetric catalysis.[11,12] Conventional Sterimol parameters quantify substituent size according to three vectors, measured along the substituent attachment axis (*L, B1*, and *B5*), **Figure *S6a***.

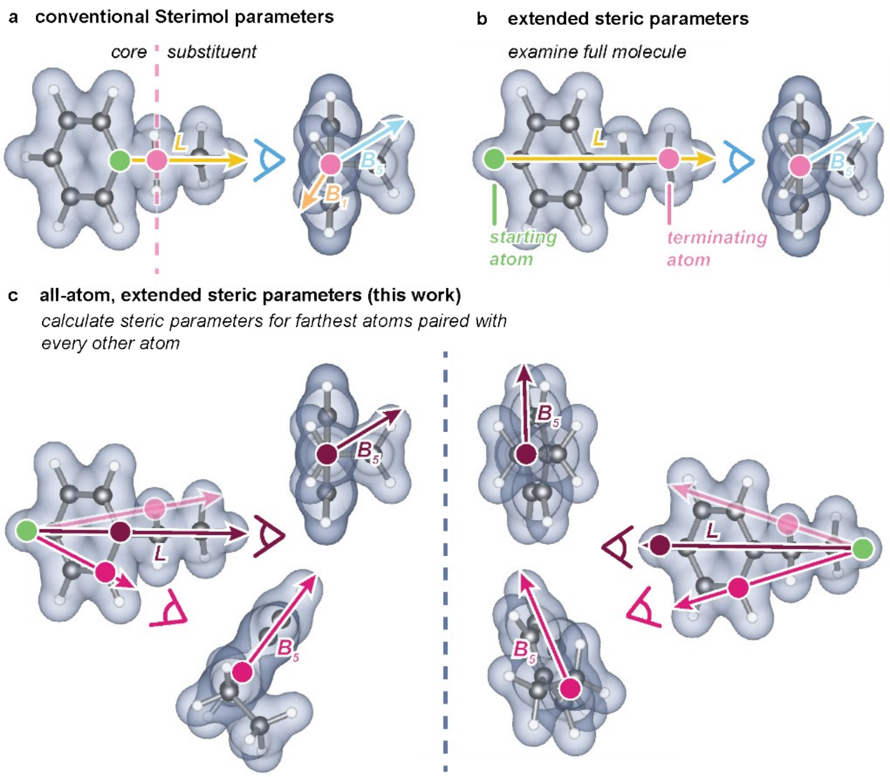

Within these studies, structure-property relationships were desired for systems when only substituent structure was varied. Within the context of PL discovery, we are concerned with quantifying the size of the entire solvent molecule. Thus, we extend the conventional Sterimol parameters to accommodate this by calculating steric parameters for vectors defined by the furthest atom paired with every other atom,

Figure S6 (a) Conventional Sterimol parameters are calculated for substituents by defining the examination vector by the atoms between the core and substituent; (b) The steric parameters for this work (SPLASHD) are extended to account for the full solvent molecule. Here, steric parameters are calculated along vectors defined by the terminal atoms (farthest atom pair) within the solvent molecule; (c) Steric parameters are calculated along vectors defined by each terminal atom with every other atom in the solvent molecule.

Figure S6b. To gain a higher resolution description of the solvent diameter distribution, we calculate extended steric parameters for all terminal atom pairs, ***Figure* S6c**; we refer to these steric parameters as "all-atom, extended steric parameters". Lastly, we are not concerned with the smallest vector, as it misrepresents the total solvent dimensions. All-atom, extended steric parameters are calculated using the SMORES software package.[13]

Considering the complex solvent-cage interaction dynamics for candidate solvent molecules that are more rigid, we must consider the rigidity of candidate solvent molecules in our size analysis. We assess rigidity using two parameters; i) number of contiguous rotatable bonds (measure of rotational axes), and ii) the steric parameter ratio for the lowest energy conformer (measure of length-to-width), ***Figure S7a***. If the candidate solvent molecule possesses ≥ 2 contiguous rotatable bonds and is 10% longer than it is wide, we consider the candidate solvent molecule to be too dynamic for a rigid size analysis. Thus, the all-atom, extended steric parameters for these systems are calculated only considering the terminal slices, ***Figures S7b***. This approach is similar to the conventional Sterimol parameter approach.[12] Rigid systems are examined as a whole, ***Figure S7c***.

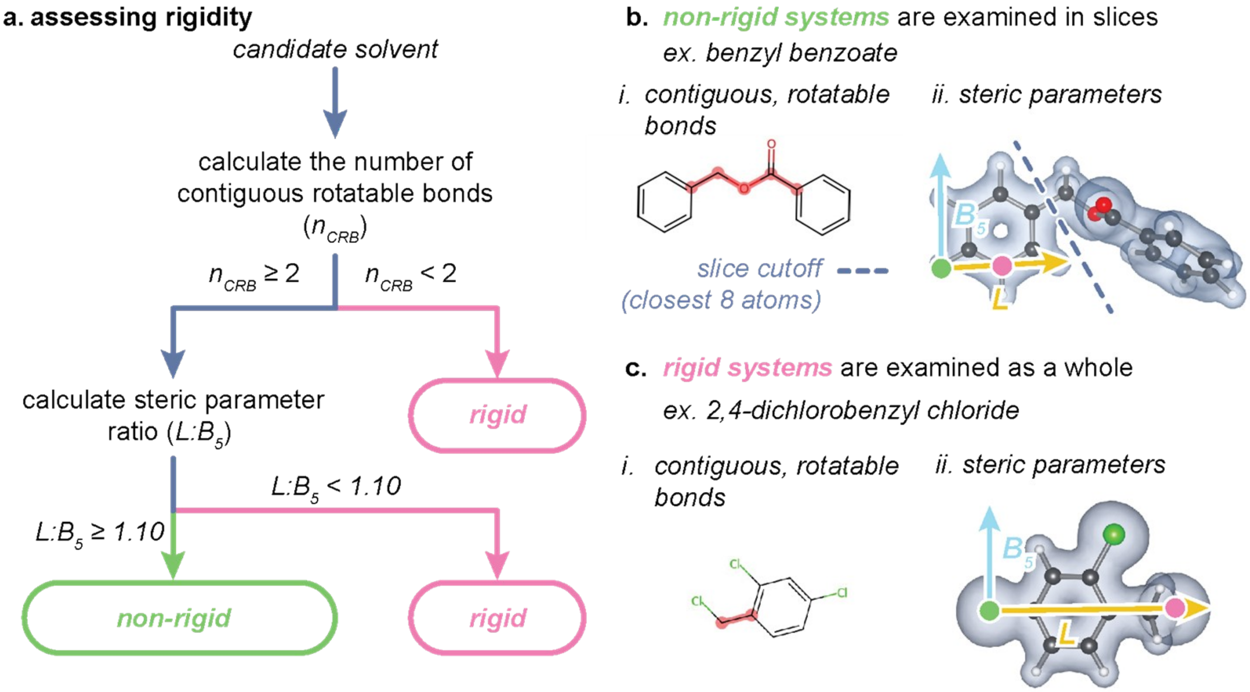

Figure S7 (a) The screening workflow used to assess the rigidity of candidate solvent molecules; (b) Rigid systems are examined in slices; benzyl benzoate is an example of a rigid system, featuring i) 5 contiguous, rotatable bonds. ii) Only the closest (by Euclidean distance) 8 atoms are included in the all-atom steric parameter calculations; (c) Rigid systems are examined as a whole; 2,4-dichlorobenzyl chloride is an example of a rigid system, featuring: (i) 1 contiguous, rotatable bond; (ii) All of the atoms are included in the all-atom steric parameter approach.

Workflow – First, a single point energy calculation is performed for each conformer extracted from the MD trajectory using *xtb*.[14–16] For the lowest energy conformer, we calculate the number of contiguous rotatable bonds and the steric parameters to determine which size analysis method should be used, ***Figure S7a***. The size analysis method is then performed for all the extracted conformers, and the corresponding steric parameters are weighted by the energy of the conformer; conformers lower in

energy contribute to the steric parameters more. This weighting procedure has recently shown promise for conventional Sterimol parameters.[11]

Size representation – From these size analysis calculations, we obtain a distribution of cage apertures, and solvent dimensions, which we plot as kernel density estimates (KDEs), main text ***Figure 2***. KDE is a non-parametric method to estimate probability density; we require a non-parametric method in this instance because there is no way to know the form that the steric parameter distribution may take. Essentially this is a probability that the chemical system will take on a particular size/dimension.

Section S5.3 Size exclusivity assessment

Table S7: Previously reported size-excluded solvents are presented in order of increasing calculated KDE overlap. Solvents are labelled as size-excluded (KDE overlap < 0.25), potentially size-excluded (0.25 < KDE overlap < 0.35), or not-size-excluded (KDE overlap > 0.35) based on the calculated KDE overlap.

Size-exclusivity prediction	Solvent	KDE overlap	Reference
size-excluded	15-crown-5	0.029	
	3,5-bistrifluoromethyl-phenol	0.036	Kearsey2019
	Hexafluoro-3-bistrifluoromethyl-buane-2,3-diol	0.158	Kearsey2019
	2,4-dichlorobenzyl-chloride	0.168	Kearsey2019
	2,4-dichlorotoluene	0.211	Kearsey2019
	2,3,5,6-tetrafluoro-4-trifluoromethylphenol	0.221	Kearsey2019
Potentially size-excluded	2'-hydroxyacetophenone	0.260	Kearsey2019
	Hexachloropropene/perchloropropene	0.264	Greenaway2017
	1,1,2,2,3,3-hexachloropropane	0.292	
	Perchloroethylene	0.349	
not size-excluded	2-fluoro-5-trifluoromethyl-phenol	0.353	Kearsey2019
	Methyl salicylate	0.355	
	4-trifluoromethoxybenzyl-alcohol	0.370	Kearsey2019
	Chloroform	0.473	
	Dichloromethane	0.629	
	Benzyl benzoate	0.712	

Section S6. Methane uptake experiments

Capillary Preparation: Approximately 0.1 mL of a solution of tetramethylsilane (TMS, 10 µL) in deuterated chloroform ($CDCl_3$, 0.5 mL) was flame sealed in a glass capillary.

Sample Preparation: The scrambled **3³:13³**-*R* cage was desolvated in a vacuum oven at 90 °C overnight prior to its use in the samples. The analyte samples were prepared by dissolving scrambled **3³:13³**-*R* cage in one of the selected solvents at the desired concentration (200 mg + 1 mL, and Table S5, using 1 mL volumetric flasks for the calibration experiments.

Capillary Calibration: Desolvated scrambled cage **CC3³:13³**-*R* (25.9 mg, 50.1 mg, 102.7 mg, 149.3 mg and 196.5 mg) was dissolved in $CDCl_3$ (0.8 mL) in a 1 mL volumetric flask before topping up to 1 mL. Each solution was transferred to an NMR tube before fitting with a $CDCl_3$/TMS capillary. ^{1}H NMR spectra were acquired for each capillary in each solution. Calibration curves were produced by plotting the ratio of the 12NC**H** and TMS signal integrations in the spectra against the concentration (Figure S10). Regression analysis was carried out using Microsoft Excel.

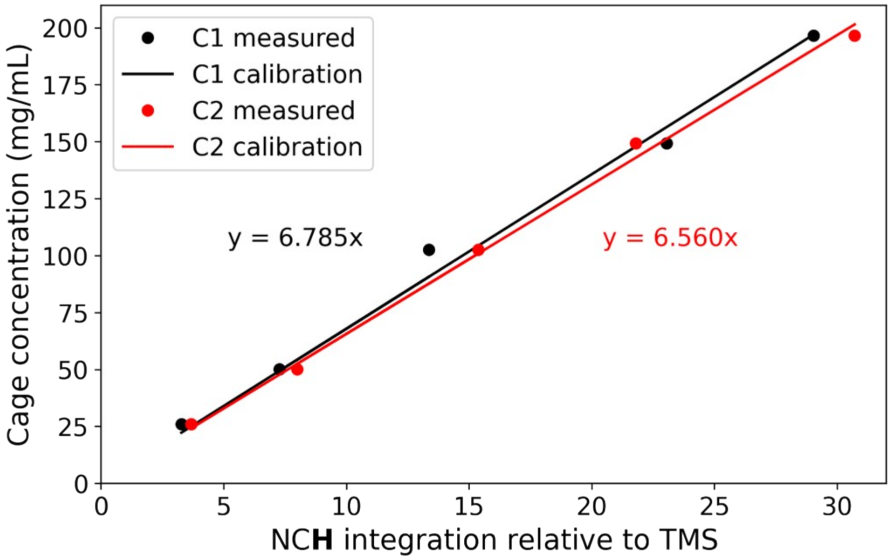

C1 Regression Statistics	
Multiple R	0.99861173
R Square	0.99722538
Adjusted R Square	0.74722538
Standard Error	7.1950024

C2 Regression Statistics	
Multiple R	0.99949148
R Square	0.99898321
Adjusted R Square	0.74898321
Standard Error	4.35557078

Figure S8: Calibration curves (left) for the 2 capillaries (C1 and C2) used in the NMR uptake experiments used in this study. Summaries of the regression analysis carried out to acquire the fits (right).

Gas uptake capacity measurements

Solutions of scrambled **3^3:13^3**-*R* were prepared in the selected solvents at either 200 mg cage + 1 mL solvent or at the saturation concentration determined in Section S4. The sample was transferred to an NMR tube fitted with a calibrated $CDCl_3$/TMS capillary and a ^{1}H NMR spectra were acquired before and after gas uptake. The same process was also carried out for the neat solvents to record the baseline solubility in the solvent.

To load the sample with methane, needle was fitted to tubing and connected to the methane gas regulator in series with a calibrated flowmeter. The assembly was purged with gas for 5 minutes at 50-60 mL/min before inserting the needle into the NMR tube and submerging the tip into the sample and bubbling at the same rate. The rate was controlled by adjusting the regulator's needle valve. Methane gas was bubbled through the sample for 10 minutes before acquiring the NMR spectra.

Of the 5 solvents selected for gas uptake measurement, the porous liquids prepared from 1-phenylethanol, 2-ethylphenol, 2-phenylpropan-1-ol and 2-methyl-1-phenylpropan-2-ol were stable to exposure to CH_4 gas, while for *o*-cresol the dissolved cage precipitated upon addition of CH_4.

The gas concentration was calculated by integrating the CH_4 signal present in the negative chemical shift range relative to TMS and using the capillaries calibration curve to calculate the concentration. For the porous liquids, increase in viscosity led to significant broadening of the solvent peaks such that it was no longer possible to integrate the TMS peak as in previous studies. Instead, a window baseline correction was applied across the region containing the TMS and CH_4 peak to deconvolute the TMS peak from the solvent resonance, allowing for integration of the TMS peak.

Table S8: CH_4 uptake capacity and associated NMR data for each of the solvents at the 200 mg cage + 1 mL solvent and at the saturation cage concentration where applicable.

Solvent	Concentration	CH_4/TMS integration	Chemical Shift	CH_4 uptake capacity	Uptake increase
1-Phenylethanol	200 mg + 1 mL	0.73	-1.37 ppm	14.3 µmol/mL	10.3 µmol/mL
2-Ethylphenol	200 mg + 1 mL	1.86	-2.28 ppm	36.4 µmol/mL	31.9 µmol/mL
2-Phenylpropan-1-ol	200 mg + 1 mL	0.73	-0.97 ppm	13.8 µmol/mL	9.7 µmol/mL
2-Methly-1-phenylpropan-2-ol	200 mg + 1 mL	1.72	-1.75 ppm	32.6 µmol/mL	26.7 µmol/mL
Diphenyl ether	200 mg + 1 mL	0.30	-0.43	5.6 µmol/mL	n/a
1-Phenylethanol	20.9% w/w	1.32	-1.78 ppm	25.8 µmol/mL	21.8 µmol/mL
2-Ethylphenol	19.9% w/w	3.04	-2.58 ppm	59.5 µmol/mL	55.0 µmol/mL
2-Phenylpropan-1-ol	19.9% w/w	1.03	-1.67 ppm	20.2 µmol/mL	15.7 µmol/mL
2-Methly-1-phenylpropan-2-ol	17.8% w/w	2.40	-2.21 ppm	47.0 µmol/mL	41.1 µmol/mL
1-Phenylethanol	Neat solvent	0.21	-0.07 ppm	4.0 µmol/mL	-
2-Ethylphenol	Neat solvent	0.23	-0.22 ppm	4.5 µmol/mL	-
2-Phenylpropan-1-ol	Neat solvent	0.21	-0.06 ppm	4.1 µmol/mL	-
2-Methly-1-phenylpropan-2-ol	Neat solvent	0.31	0.01 ppm	5.9 µmol/mL	-

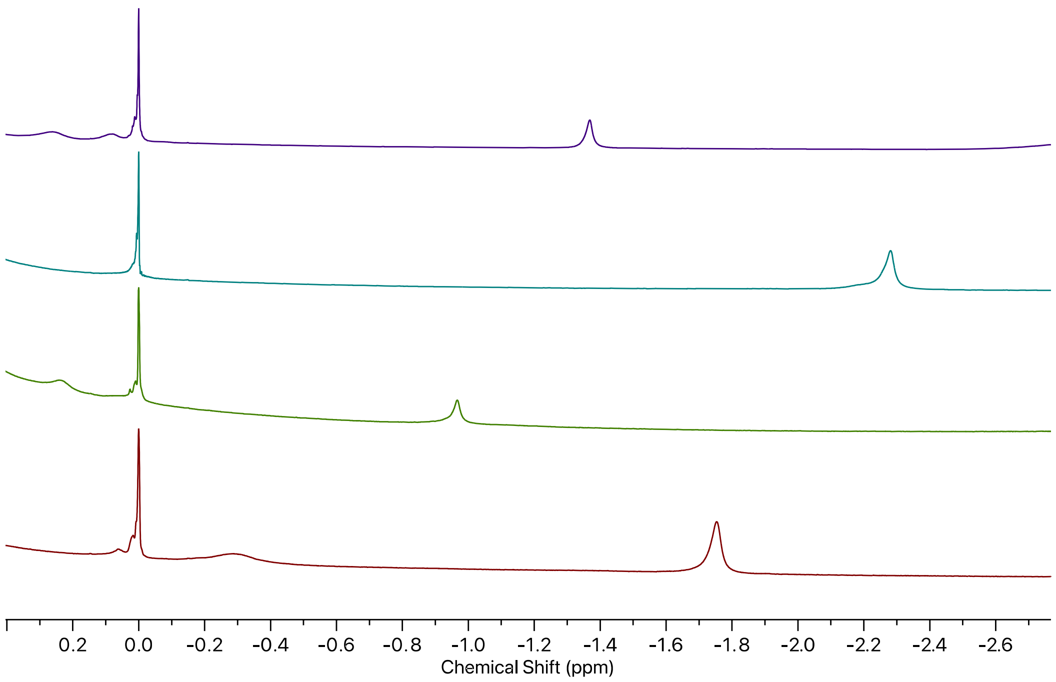

Figure S9 Stacked ^{1}H NMR spectra of the CH_4 saturated porous liquid samples prepared at 200 mg + 1 mL concentration in 1-phenylethanol (top), 2-ethyl phenol (second from top), 2-phenylpropan-2-ol (second from bottom) and 2-methyl-1-phenylpropan-2-ol (bottom).

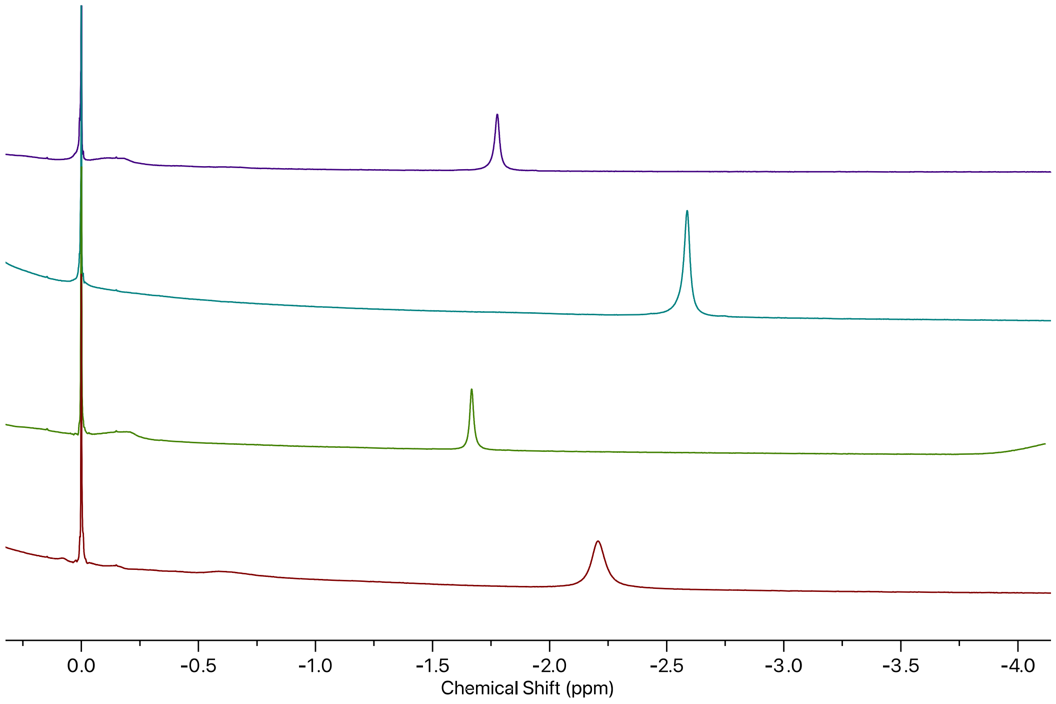

Figure S10 Stacked ^{1}H NMR spectra of the CH_4 saturated porous liquid samples prepared at the maximum possible concentration in 1-phenylethanol (top), 2-ethyl phenol (second from top), 2-phenylpropan-2-ol (second from bottom) and 2-methyl-1-phenylpropan-2-ol (bottom).

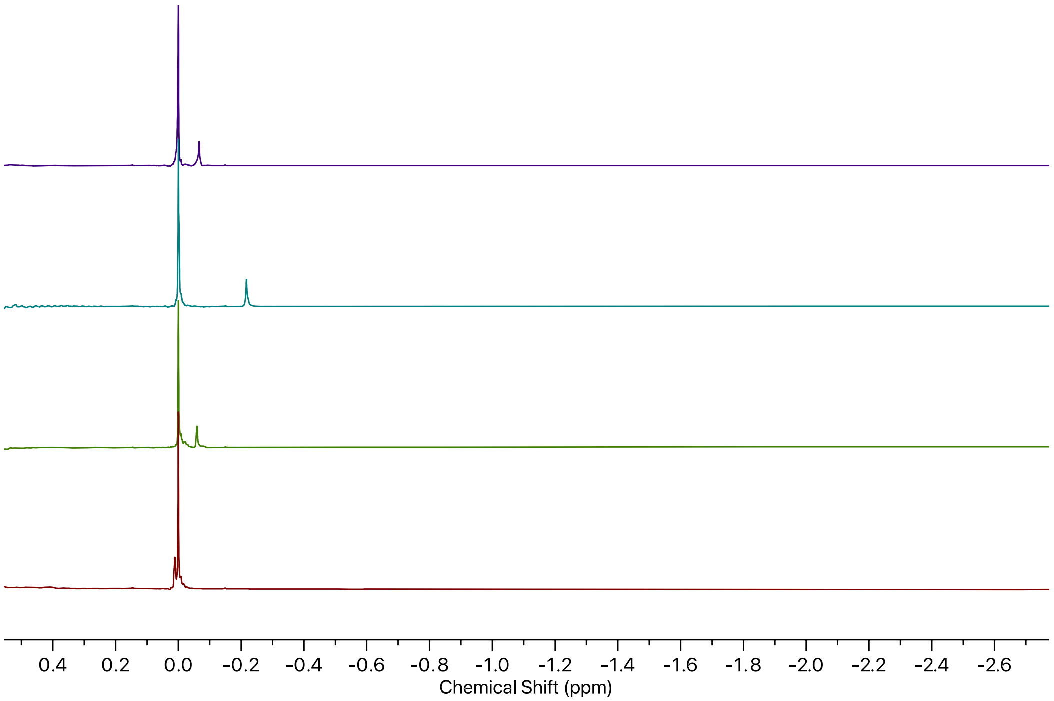

Figure S11 Stacked ^{1}H NMR spectra of the CH_4 saturated solvents 1-phenylethanol (top), 2-ethyl phenol (second from top), 2-phenylpropan-2-ol (second from bottom) and 2-methyl-1-phenylpropan-2-ol (bottom).

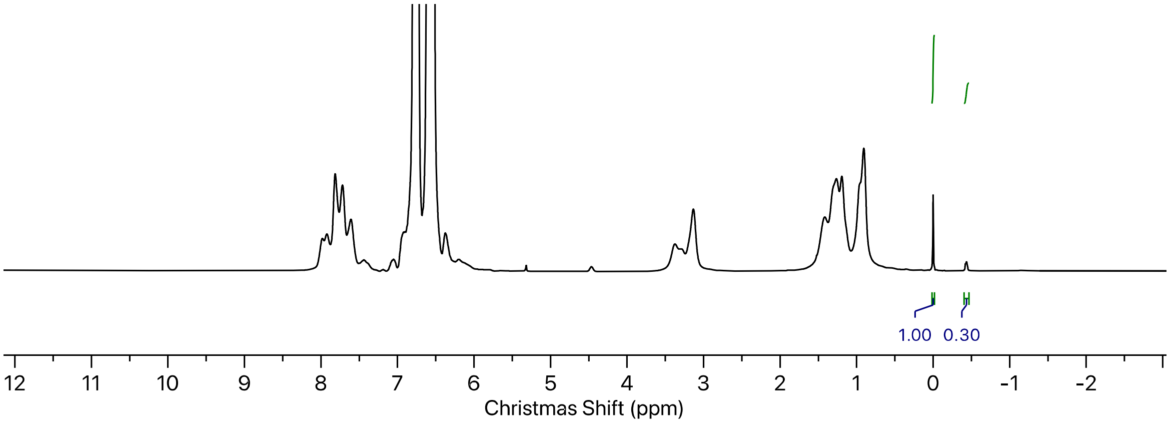

Figure S12 ^{1}H NMR spectrum of the solution of **3³:13³**-*R* scrambled cage mixture in diphenyl ether saturated with CH_4 gas.

References

1 R. L. Greenaway, D. Holden, E. G. B. Eden, A. Stephenson, C. W. Yong, M. J. Bennison, T. Hasell, M. E. Briggs, S. L. James and A. I. Cooper, *Chem. Sci.*, 2017, **8**, 2640–2651.

2 L. Turcani, A. Tarzia, F. T. Szczypiński and K. E. Jelfs, *J. Chem. Phys.*, 2021, **154**, 214102.

3 E. Harder, W. Damm, J. Maple, C. Wu, M. Reboul, J. Y. Xiang, L. Wang, D. Lupyan, M. K. Dahlgren, J. L. Knight, J. W. Kaus, D. S. Cerutti, G. Krilov, W. L. Jorgensen, R. Abel and R. A. Friesner, *J. Chem. Theory Comput.*, 2016, **12**, 281–296.

4 F. Mohamadi, N. G. J. Richards, W. C. Guida, R. Liskamp, M. Lipton, C. Caufield, G. Chang, T. Hendrickson and W. C. Still, *J. Comput. Chem.*, 1990, **11**, 440–467.

5 K. S. Watts, P. Dalal, A. J. Tebben, D. L. Cheney and J. C. Shelley, *J. Chem. Inf. Model.*, 2014, **54**, 2680–2696.

6 stko https://github.com/JelfsMaterialsGroup/stko.

7 A. Kai, A. Mroz, K. E. Jelfs, A. I. Cooper, M. A. Little and R. L. Greenaway, *Mol. Syst. Des. Eng.*, 2025, 10.1039.D5ME00004A.

8 J. M. Rimsza and T. M. Nenoff, *J. Mol. Liq.*, 2024, **401**, 124731.

9 M. Miklitz and K. E. Jelfs, *J. Chem. Inf. Model.*, 2018, **58**, 2387–2391.

10 A. Verloop, W. Hoogenstraaten, J. Tipker, B. V. Philips-Duphar and N. Weesp, *Drug Des.*

11 A. V. Brethomé, S. P. Fletcher and R. S. Paton, *ACS Catal.*, 2019, **9**, 2313–2323.

12 K. C. Harper, E. N. Bess and M. S. Sigman, *Nat. Chem.*, 2012, **4**, 366–374.

13 A. M. Mroz, L. Turcani and K. E. Jelfs, *Electron. Struct.*, 2023, **5**, 045004.

14 C. Bannwarth, S. Ehlert and S. Grimme, *J. Chem. Theory Comput.*, 2019, **15**, 1652–1671.

15 C. Bannwarth, E. Caldeweyher, S. Ehlert, A. Hansen, P. Pracht, J. Seibert, S. Spicher and S. Grimme, *WIREs Comput. Mol. Sci.*, 2021, **11**, e1493.

16 S. Grimme, C. Bannwarth and P. Shushkov, *J. Chem. Theory Comput.*, 2017, **13**, 1989–2009.
